# Inpatient and postdischarge mortality among children with anaemia and malaria parasitaemia in Kenya: a cohort study

**DOI:** 10.1136/bmjgh-2024-016600

**Published:** 2024-10-21

**Authors:** Moses Ngari, Martha Mwangome, Nelson Ouma, Amek Nyaguara, Neema Mturi, Christina Obiero, Alice Kamau, Judd L Walson, Per O Iversen, Kathryn Maitland, Robert W Snow, James Berkley

**Affiliations:** 1 KEMRI-Wellcome Trust Research Programme, Kilifi, Kenya; 2 The Childhood Acute Illness and Nutrition (CHAIN) Network, Nairobi, Kenya; 3 KEMRI Centre for Geographic Medical Research, Coast (CGMR(C), Kilifi, Kenya; 4 EDD, KEMRI-Wellcome Trust Research Programme, Kilifi, Kenya; 5 Department of Global Health, University of Amsterdam, Amsterdam, The Netherlands; 6 KEMRI-Wellcome Trust Research Programme Nairobi, Nairobi, Kenya; 7 Department of International Health, Johns Hopkins University, Baltimore, Virginia, USA; 8 University of Oslo Institute of Basic Medical Sciences, Oslo, Norway; 9 Oslo University Hospital, Oslo, Norway; 10 Imperial College London Department of Medicine, London, UK; 11 Centre for Tropcial Medicine and Global Health, Oxford University, Oxford, UK

**Keywords:** Child health, Paediatrics, Anaemia, Malaria, Global Health

## Abstract

**Background:**

Anaemia and malaria are leading causes of paediatric hospitalisation and inpatient mortality in sub-Saharan Africa. However, there is limited empirical data on survival following hospital discharge. We aimed to estimate independent effects of anaemia and malaria parasitaemia on inpatient and 1 year postdischarge mortality among Kenyan children.

**Methods:**

A retrospective cohort study among children admitted to Kilifi County Hospital (KCH) from 2010 to 2019 and followed-up for 1 year postdischarge in Kilifi Health and Demographic Surveillance System (KHDSS). The main exposures were anaemia and malaria parasitaemia at the time of hospital admission while inpatient and 1 year postdischarge mortality were the outcomes.

**Results:**

We included 9431 admissions among 7578 children (43% girls), median (IQR) age 19 (9.9‒23) months. 2069 (22%), 3893 (41%) and 1140 (12%) admissions had mild, moderate and severe anaemia, whereas 366 (3.9%), 779 (8.3%) and 224 (2.4%) had low, medium and high malaria parasitaemia, respectively. Overall, there were 381 (4.0%) inpatient deaths: 317/381 (83%) and 47/381 (12%) among children with any level of anaemia and malaria parasitaemia, respectively. Moderate and severe, but not mild anaemia, were positively associated with inpatient death. Low and high level parasitaemia were positively associated with inpatient mortality, while medium level parasitaemia was negatively associated. There were 228 (3.1%) postdischarge deaths: 32.8 (95% CI 28.8‒37.3) deaths/1000 child-years. 180/228 (79%) deaths occurred within 6 months after index discharge and 99/228 (43%) occurred in the community. Overall, 180/228 (79%) and 10/228 (4.4%) postdischarge deaths occurred among children with any level of anaemia and malaria parasitaemia, respectively. Severe anaemia was positively associated with postdischarge mortality (adjusted HR 1.94 (95% CI 1.11‒3.40)), while medium level parasitaemia was negatively associated.

**Conclusion:**

Interventions to create awareness of postdischarge risks, improve uptake of existing interventions and improved discharge processes targeting high-risk groups such as children admitted with severe anaemia, need to be prioritised.

**WHAT IS ALREADY KNOWN ON THIS TOPIC**

- Anaemia and malaria are leading causes of paediatric hospitalisation and inpatient mortality in sub-Saharan Africa.
- Mortality following discharge from hospital in resource-poor countries has been recognised as a significant contributor to childhood mortality. However, there is limited empirical data on survival following hospital admission distinguishing the effects of anaemia and malaria parasitaemia.

**WHAT THIS STUDY ADDS**

- Inpatient mortality was higher among children admitted with severe anaemia (7.7%) or high malaria parasitaemia (6.3%) than overall inpatient deaths (4.0%). However, during 1 year postdischarge, children admitted with high malaria parasitaemia had very low mortality (1.0%) regardless of the presence of anaemia, but those admitted with severe anaemia had higher mortality (4.3%) than all children discharged (3.1%).
- Admission with severe anaemia but not high malaria parasitaemia was associated with high risk of postdischarge mortality.

**HOW THIS STUDY MIGHT AFFECT RESEARCH, PRACTICE OR POLICY**

- Recognising high-risk children at discharge such as those with severe anaemia and appropriate continuity of care in the community/outpatient need to be prioritised.

## Background

 Anaemia and malaria are leading antecedents of paediatric hospitalisation, morbidity and mortality in many low-income and middle-income countries (LMICs)^1^ with children under 5 years of age bearing most risk.^2^ In malaria-endemic areas, severe anaemia commonly accounts for up to a third of child admissions and has a multifactorial aetiology.^3^ Severe anaemia is associated with significant inpatient^3 4^ and postdischarge mortality.^4^,^7^ Several interventions have been shown to reduce all-cause mortality among children following admission with anaemia and/or malaria, including artesunate-based malaria treatment, postdischarge malaria chemoprophylaxis and adherence to standardised protocols.^8^ However, neither an enhanced volume of immediate blood transfusion, micronutrient supplementation nor antimicrobial prophylaxis reduced postdischarge mortality among children with severe anaemia.^9 10^

Postdischarge mortality occurs across all major clinical syndromes.^11^ In LMICs, the number of child deaths during 6 months postdischarge is similar to those occurring in hospital.^6 11 12^ In a recent systematic review, the pooled 6 month postdischarge mortality among children admitted with severe anaemia (6.4%) was higher than admissions with general acute illness (4.4%).^4 11^ Anaemia was associated with recurrent admission, with 17.3% readmitted within 6 months after discharge, threefold higher than children admitted with other conditions.^4^ Conversely, postdischarge mortality is reportedly lower among children admitted with malaria (2.8%) than children admitted for general acute illness (4.4%).^11^ However, prior studies have had diverse study designs, inconsistent diagnostic criteria, varying duration of follow-up and sometimes a high loss to follow-up.^11^ Definitions of severe anaemia have varied with cutoffs of haemoglobin <5 to <7 g/L and for malaria, from clinically suspected malaria to positive blood slide for parasites or rapid diagnostic test for *Plasmodium falciparum* without necessarily considering that parasitaemia may be coincidental rather than the cause of illness.^11 13^ Thus, the relationships between different levels of anaemia and malaria parasitaemia, other comorbidities and mortality are unclear. Despite potential mortality risks, currently there are limited international and national guidelines on targeted postdischarge care.^11 12 14 15^

We aimed to describe a large cohort of Kenyan children under 5 years of age admitted to hospital with anaemia and/or malaria parasitaemia and estimate their inpatient and 1 year postdischarge mortality.

## Methods

### Study setting

This study was conducted within the Kilifi Health and Demographic Surveillance System (KHDSS), located in coastal Kenya, including children admitted to Kilifi County Hospital (KCH). KCH is a level IV hospital offering both outpatient and inpatient care with two paediatric wards; a general ward with 70 beds and a high dependency unit with 15 beds managed by research nurses and clinicians. Children are managed according to Kenya national and WHO guidelines. From 2010 to date, children admitted with moderate or severe malaria at KCH were treated with parenteral artesunate, then when able to take oral medicines and the non-severe, artemether/lumefantrine.^16^ Blood transfusion was prescribed to eligible children according to WHO guidelines, however blood transfusion units were sometimes not promptly available.^17 18^ Children with anaemia not requiring blood transfusion were managed for comorbidities and other underlying conditions while monitoring the haemoglobin levels. At hospital discharge, children needing postdischarge care such as anthelmintics, iron and folate recommended by WHO are linked to outpatient clinics offering these services.^19^

**Figure 1:**
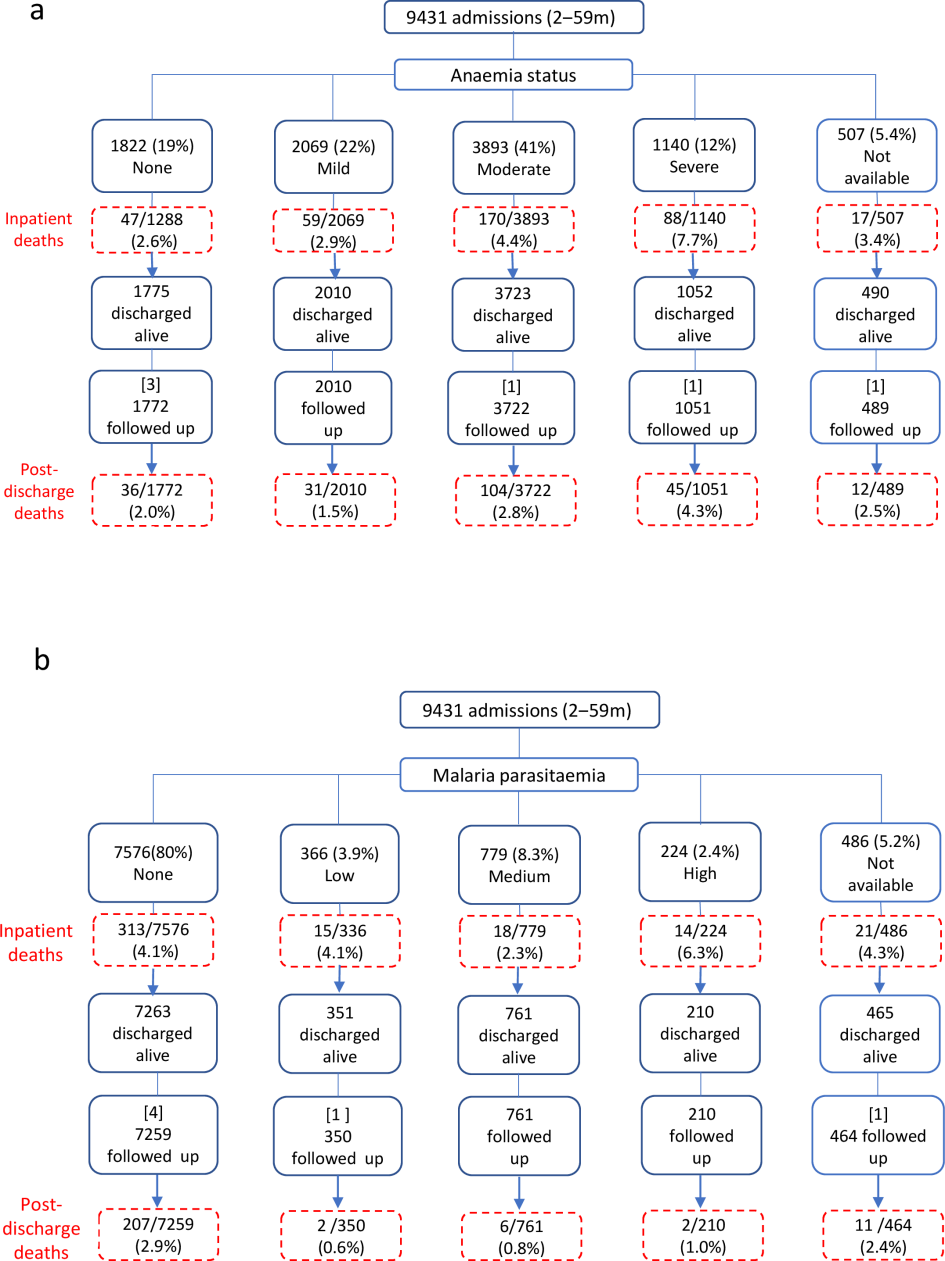
Study participants flow stratified by (a) anaemia levels and (b) malaria parasitaemia density. In panel (**a**), 3/1775 (0.17%), 1/3723 (0.03%), 1/1052 (0.10%) and 1/490 (0.20%) were missing follow-up data among admissions with none, moderate, severe and with no available anaemia status data, respectively. In panel (**b**), 4/7263 (0.06%), 1/351 (0.27%) and 1/465 (0.21%) were missing follow-up data among admissions with none, low and no available malaria parasitaemia, respectively. All the records missing follow-up data are shown in square brackets ([ ]) in the figures.

At admission, data on anthropometry, clinical signs, laboratory investigations including a malaria parasite slide and complete blood count have been systematically recorded since 1998. Data were routinely collected by experienced research clinical teams. Diagnostic test kits for anaemia and malaria parasitaemia were provided by the research institute and were rarely out of stock. In 2002, the KHDSS was established to collect community-based data on births, migration, pregnancies and deaths among approximately 300 000 people who reside within 891 km^2^ south and north of Kilifi Town (online supplemental methods and figure 1).^20^ Malaria transmission is much higher in the southern than northern area and transmission has a seasonal pattern, peaking during the long (April to June) and short (October to December) rain periods (online supplemental methods).^21 22^ The population is enumerated every 4 months, and these data are linked to the KCH admissions through unique identifiers.

### Study design

We conducted a retrospective cohort study. The outcomes of interest were both inpatient and 1 year postdischarge mortality within the KHDSS. The main exposures were malaria parasitaemia and anaemia at the time of hospital admission.

### Study population

We included data from children aged 2–59 months at the time of admission to KCH and resident within the KHDSS admitted from 1 January 2010 to 31 December 2019. Postdischarge follow-up included children’s data in the KHDSS 1 year after hospital discharge meaning the last child was followed up to 31 December 2020. Children admitted to KCH, but resident outside the KHDSS, were excluded because they lacked follow-up data after hospital discharge.

### Data sources

A standardised form was used to collect data on medical history, clinical examination and anthropometry by research-trained clinical staff (online supplemental methods). Blood samples for malaria microscopy, complete blood cell count, blood culture and HIV antibody test were systematically collected at admission before treatment was started although some children could have taken antimalarials before admission. Giemsa-stained thick and thin blood films were examined for *P. falciparum* parasites according to standard methods and data were presented as malaria parasite density (MPD), that is, the number of infected red blood cells (RBCs) per 500, 200 or 100 RBC total counted. Parasitaemia per litre of blood was calculated as (the number of parasitised RBCs × number of RBCs per litre)/(number of RBCs counted or number of parasites counted times the number of white cell counts (WCCs) per litre/number of WCCs counted) online supplemental methods.^16 23^ Sickle cell genotyping was not routinely undertaken, but was recorded as a discharge diagnosis from parental reporting or testing done during admission. On discharge from the hospital or inpatient death, the clinician assigned up to two discharge diagnosis and recorded if a blood transfusion had been ordered. Causes of deaths in the community within KHDSS have been determined using verbal autopsy since 2008 (online supplemental methods).^24^

### Clinical definitions

Anaemia was classified per WHO definitions: (a) none (haemoglobin ≥11 g/L), (b) mild (haemoglobin 10‒10.9 g/L), (c) moderate (haemoglobin 7‒9.9 g/L) and (d) severe (haemoglobin<7 g/L).^25^ MPD was categorised into four groups: (a) none, (b) low (<2500/L), (c) medium (2500‒250 000/L) and (d) high (>250 000/L).^13^ Definition of other clinical diagnosis following WHO guidelines is provided in online supplemental methods.^19^

### Study size

All admissions to KCH during study period and residents within KHDSS were included (n=9431). This study size had power greater than 90% to show a HR of 1.5 or more for severe anaemia on postdischarge mortality assuming at least 30 deaths/1000 child-years and a two-tailed alpha of 5%.

### Quantitative variables

Anthropometric z scores were calculated using 2006 WHO growth standards.^26^ We report wasting defined using the middle upper arm circumference (MUAC) because MUAC is less affected by dehydration compared with weight-based anthropometry,^27^ easier to measure than length-based indices in sick children and had the least missing records. Wasting was classified in three groups: (a) not wasted (MUAC ≥12.5 cm for those aged ≥6 months or MUAC ≥12.0 cm for <6 months old), (b) moderately wasted (MUAC 11.5‒12.5 cm for those aged ≥6 months or MUAC 11‒12.0 cm for <6 months old) and severely wasted (MUAC<11.5 cm for those aged ≥6 months or MUAC<11.0 cm for <6 months old or kwashiorkor at any age).^6^

### Statistical analysis

The proportion of data missing in all variables was low (<5%) and was assumed not to be missing at random and a classification of ‘not available’ was included in the analyses. Results are reported stratified by anaemia and malaria parasitaemia status. All analyses used the number of admissions rather than number of children because some children had more than one admission during the study period.

For inpatient mortality, we treated being discharged alive as a competing risk with death and used the Fine-Gray proportional subdistribution hazards model accounting for the multiple admissions per child.^28^ Time at risk was from date of admission to date of death or discharge. We report the adjusted subdistribution Hazard Ratios (aSHR) from the regression models with 95% CI. The univariate models included either anaemia or malaria parasitaemia as the only independent variables. The multivariable model included both anaemia and malaria parasitaemia categorical variables, adjusted for the following confirmed confounders: age, nutritional status, HIV, known sickle cell disease, cerebral palsy, heart disease, epilepsy, bacteraemia, year of admission, season (rainy/dry), KHDSS area (South, North or Kilifi Township) since the South is known to have higher malaria endemicity^21^ and blood transfusion during admission.

**Figure 2:**
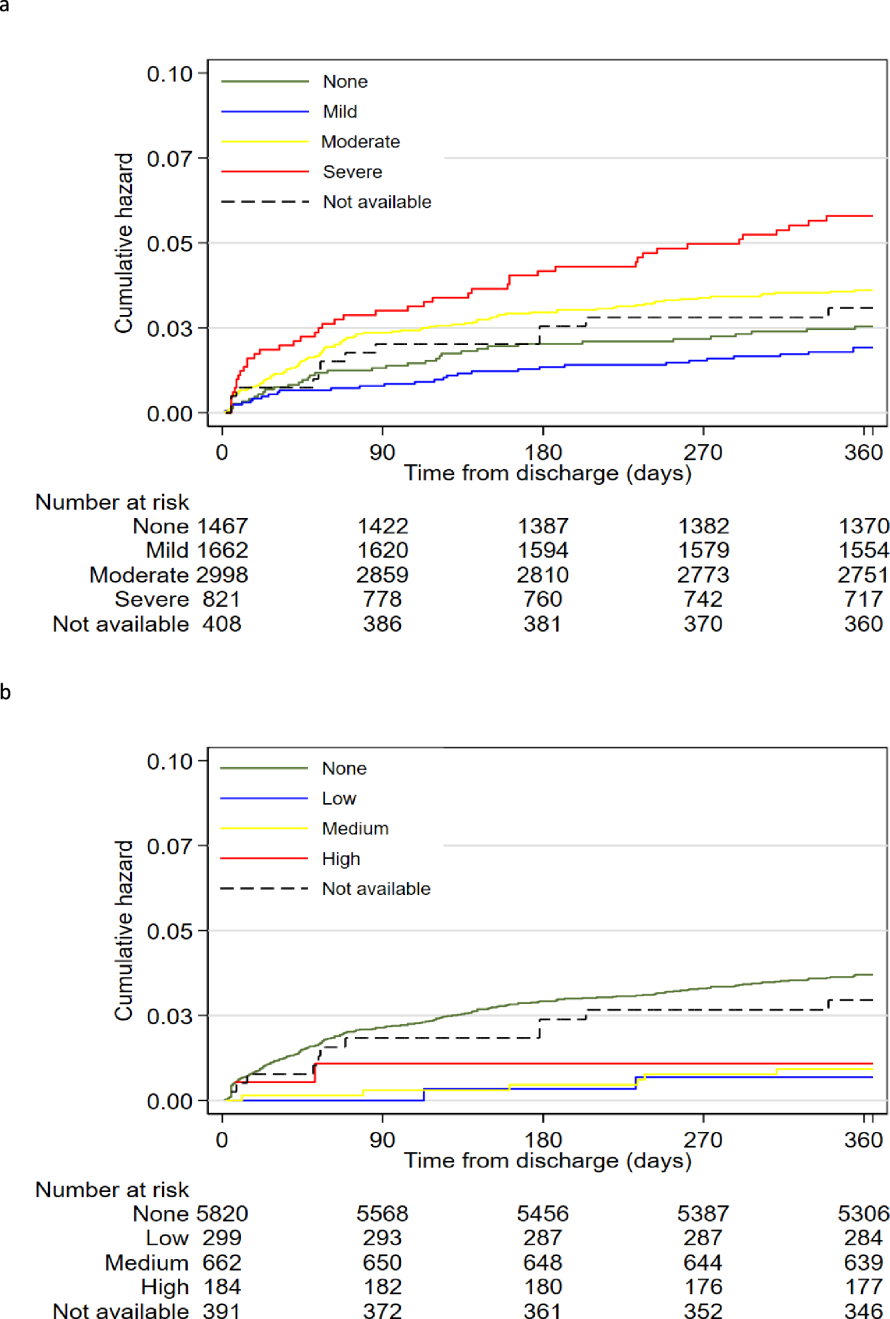
Cumulative hazards of postdischarge mortality stratified by (**a**) anaemia levels and (**b**) malaria parasitaemia density.

The postdischarge analysis included children who were discharged alive. Time at risk of postdischarge death was from the date of discharge to 365 days later, date of death or outmigration from KHDSS. We performed postdischarge analysis including multiple admissions per child resetting time at risk at every live discharge. Any death after index discharge within 365 days including those occurred during readmissions were included. Schoenfeld residuals were used to test the proportional hazards assumption for both inpatient and postdischarge analysis (no violation found). To explicitly account for the multiple admissions per child, we used a multilevel mixed-effects parametric survival models including the child ID as the random intercept. Weibull distribution was the best fitting probability distribution (online supplemental methods, table 1, figures 2 and 3). We performed both univariate and multivariable multilevel mixed-effects parametric survival regression analysis with Weibull distribution and report Hazard Ratios (HR as the measure of effect. The multivariable model was adjusted for the same confounders as the inpatient multivariable model.

We also estimated whether blood transfusion modified the risk of death postdischarge by comparing models with and without an interaction term between anaemia or MPD and blood transfusion using likelihood ratio tests (noting that haemoglobin levels may have changed during admission later prompting transfusion). To estimate the overall effect of blood transfusion on the risk of postdischarge death, we estimated effects on each anaemia classification and used random-effects meta-analysis approach to pool the effects across the levels of anaemia and of parasitaemia at admission. All statistical analyses were conducted using R software V.4.2.0 and STATA V.17.0 (College Station, TX, USA).

### Ethical considerations

This analysis was approved by the Kenya Medical Research Institute (KEMRI) Scientific and Ethics Review Unit (SCC 2778). Caregivers of the study participants provided written consent to participate in the hospital surveillance and KHDSS. We report the data according to the STrengthening the Reporting of Observational Studies in Epidemiology (STROBE) guidelines (online supplemental methods: STROBE statement).

### Patients and public involvement

Patients and public were not involved in the design, conduct and reporting of this research.

## Results

There were 18 913 potentially eligible admissions, of which 9431 (50%) were residents within KHDSS and thus included in the study. The 9431 admissions occurred among 7578 children. The median age (IQR) of admissions was 19 (9.9‒34) months and 4044 (43%) were female. Overall, 874 (9.3%) admissions had moderate wasting and 842 (8.9%) severe wasting, while 322 (3.4%) had kwashiorkor. Difficulty breathing (n=3075, 33%) and lower chest indrawing (n=2685, 28%) were common clinical signs at admission. Severe pneumonia (n=2924, 31%) and diarrhoea (n=1856, 20%) were common admission codiagnoses. HIV and sickle cell disease were diagnosed in 267 (2.8%) and 414 (4.4%) admissions, respectively (table 1). All admission characteristics stratified by anaemia levels and malaria parasitaemia density are shown in online supplemental table 2a. The admission characteristics of the 9482 non-KHDSS residents are shown in online supplemental table 2b. Severe clinical conditions were more frequent among the non-KHDSS than KHDSS residents (online supplemental table 3).

**Table 1:** Children characteristics at admission to hospital

Characteristics (N=9431)	No (%)
Sex—female	4044 (43)
Age in months	
<6	1231 (13)
6‒11	1684 (18)
12‒23	2678 (28)
≥24	3838 (41)
Median days of hospital stay (IQR)	
Survivors	3 (2‒6)
Deaths	2 (1‒6)
Received blood transfusion	488 (5.2)
Repeated admissions	1782 (19)
KHDSS region	
North	2752 (29)
Kilifi Township	2409 (26)
South	4270 (45)
Season of the year	
Dry	4786 (51)
Rainy	4645 (49)
Nutritional status	
Not wasted	7653 (81)
Moderately wasted	874 (9.3)
Severely wasted	842 (8.9)
Missing MUAC	62 (0.7)
Kwashiorkor	322 (3.4)
Clinical signs	
Axillary temperature	
<36°C	529 (5.6)
36°C to 37.5°C	4041 (43)
>37.5°C to 39°C	3463 (37)
>39°C	1390 (15)
Missing	8 (0.08)
Respiratory rate/min	
Bradypnoea	377 (4.0)
Normal	5912 (63)
Tachypnoea	3090 (33)
Missing	52 (0.6)
Heart rate/min	
Bradycardia	166 (1.8)
Normal	4419 (47)
Tachycardia	4842 (51)
Missing	4 (0.04)
Hypoxia	577 (6.1)
Breathing difficulty	3075 (33)
Lower chest indrawing	2685 (28)
Wheeze	521 (5.5)
Capillary refill>2 s	238 (2.5)
Weak pulse	194 (2.1)
Sunken eyes	1012 (11)
Reduced skin turgor	473 (5.0)
Dehydration status	
No dehydration	8283 (88)
Some dehydration	488 (5.2)
Severe dehydration	660 (7.0)
Impaired consciousness	997 (11)
Convulsion	2247 (24)
Pallor	1882 (20)
Diagnosis/laboratory features	
Severe pneumonia	2924 (31)
Diarrhoea	1856 (20)
HIV status	267 (2.8)
Bacteraemia	436 (4.6)
Epilepsy	267 (2.8)
Heart disease	174 (1.8)
Sickle cell disease	414 (4.4)
Cerebral palsy	92 (1.0)
Anaemia	
None	1822 (19)
Mild	2069 (22)
Moderate	3893 (41)
Severe	1140 (12)
Not available	507 (5.4)
Malaria parasitaemia	
None	7576 (80)
Low	366 (3.9)
Medium	779 (8.3)
High	224 (2.4)
Not available	486 (5.2)

Low malaria parasitaemia; <2500/mL, medium malaria parasitaemia; 2500–250 000/mL, high malaria parasitaemia; >250 000/mL, no anaemia; haemoglobin ≥11g/L, mild anaemia; haemoglobin 10–10.9 g/L, moderate anaemia; haemoglobin 7–9.9 g/L, severe anaemia; haemoglobin <7g/L, bradypnoea; respiratory rate <30/min for those aged <12, <24 for those aged 12–<36 and <22 for those aged ≥36 months, normal respiratory rate; respiratory rate 30–60 for those aged <12, 24–40 for those aged 12 to <36 and 22–34 for those aged ≥36 months, tachypnoea; respiratory rate >60 for those aged <12, >40 for those aged 12–<36 and >34 for those aged ≥36 months, bradycardia; heart rate<100/min for those aged <12, <90 for those aged 12–<36 and <80 for those aged ≥36 months, normal heart rate; heart rate 100–160 for those aged <12, 90–150 for those aged 12 to <36 and 80–140 for those aged ≥36 months, tachycardia; heart rate >160 for those aged <12, >150 for those aged 12 to <36 and >140 for those aged ≥36 months, rainy season is from April to June and October to December.

KHDSS, Kilifi Health Demographic Surveillance System; MUAC, middle upper arm circumference.

### Admissions with anaemia

There were 2069 (22%), 3893 (41%) and 1140 (12%) admissions with mild, moderate and severe anaemia, respectively (figure 1a). Nineteen (0.9%), 61 (1.6%) and 384 (34%) admissions with mild, moderate and severe anaemia at admission, respectively, received a blood transfusion.

### Admissions with malaria parasitaemia

There were 366 (3.9%), 779 (8.3%) and 224 (2.4%) admissions with low, medium and high MPD, respectively (figure 1b). Two hundred and sixty-eight (73%), 536 (69%) and 158 (71%) admissions with low, medium and high MPD, respectively, were ≥24 months old (table 1 and online supplemental figure 4).

A diagnosis of severe pneumonia was more frequent in admissions with low (17%) than medium (14%) and high (12%) MPD (p value<0.001). Severe malnutrition (SM), HIV infection, epilepsy, heart disease, sickle cell disease and cerebral palsy (all p values<0.001) were more frequent among admission with severe anaemia compared with high MPD.

### Admissions with malaria parasitaemia and anaemia concurrently

Among admissions with low, medium and high MPD, 100 (27%), 228 (29%) and 39 (17%) had severe anaemia, respectively, at admission (online supplemental table 2a). Among admissions with high MPD, 52 (23%) had mild and 104 (46%) moderate anaemia.

### Inpatient deaths

Overall, there were 381 (4.0%, 95% CI 3.7‒4.5) inpatient deaths: 47/1822 (2.6%), 59/2069 (2.9%), 170/3893 (4.4%) and 88/1140 (7.7%) among children with no, mild, moderate and severe anaemia, respectively. Admissions with any level of anaemia constituted 83% (317/381) of all inpatient deaths (figure 1a). We found no significant difference in time to inpatient death among children with severe anaemia by blood transfusion status; median (IQR) days 4 (3‒6) versus not transfused 4 (2‒7) (p=0.06). However, children requiring blood transfusion but did not receive blood had higher risk of inpatient death 18% compared with those transfused 6.8%, p value<0.001. There were 313/7576 (4.1%), 15/336 (4.1%), 18/779 (2.3%) and 14/224 (6.3%) deaths among children with no, low, medium and high MPD, respectively. Admissions with any level of MPD constituted 12% (47/381) of all inpatient deaths (figure 1b and online supplemental table 4). The risk of inpatient death was consistently high among children with severe anaemia and any level of MPD (table 2). In a sensitivity analysis including only the first admission (excluding readmissions), the risk of inpatient death was 288 (3.8%, 95% CI 3.3‒4.2) online supplemental table 5.

**Table 2:** Inpatient and postdischarge deaths across combinations of anaemia levels and malaria parasitaemia

Anaemia	Inpatient deaths						Postdischarge deaths					
	Malaria parasitaemia					All N (381)	Malaria parasitaemia					All (n=228)
	None (n=313)	Low (n=15)	Medium (n=18)	High (n=14)	Not available (n=21)		None (n=207)	Low (n=2)	Medium (n=6)	High (n=2)	Not available (n=11)	
None	45 (2.8)	1 (2.0)	0	1 (3.6)	0	47 (2.6)	35 (2.7)	0	1 (1.0)	0	0	36 (2.5)
Mild	56 (3.1)	0	0	2 (3.8)	1 (5.0)	59 (2.9)	31 (2.2)	0	0	0	0	31 (1.7)
Moderate	146 (4.4)	9 (5.8)	6 (2.1)	6 (5.8)	3 (6.8)	170 (4.4)	101 (4.0)	0	2 (0.8)	1 (1.2)	0	104 (3.5)
Severe	65 (8.6)	5 (5.0)	12 (5.3)	5 (13)	1 (7.1)	88 (7.7)	38 (7.6)	2 (2.2)	3 (1.6)	1 (3.1)	1 (11)	45 (5.5)
Not available	1 (1.0)	0	0	0	16 (4.1)	17 (3.4)	2 (2.5)	0	0	0	10 (3.1)	12 (3.0)

Low malaria parasitaemia; <2500/mL, medium malaria parasitaemia; 2500–250 000/mL, high malaria parasitaemia; >250 000/mL, no anaemia; haemoglobin ≥11g/L, mild anaemia; haemoglobin 10–10.9 g/L, moderate anaemia; haemoglobin 7–9.9 g/L, severe anaemia; haemoglobin <7g/L.

Moderate (aSHR 1.38 (95% CI 1.12‒1.69)) and severe anaemia (aSHR 1.94 (95% CI 1.71‒2.20)), but not mild anaemia, were positively associated with inpatient death (table 3). MPD had a U-shaped association with inpatient death: low (aSHR 1.20 (95% CI 113‒1.27)), medium (aSHR 0.67 (95% CI 0.63‒0.72)) and high MPD (aSHR 2.37 (95% CI 2.00‒2.80)) compared with no parasitaemia (table 3).

**Table 3:** Anaemia levels and malaria parasitaemia density and all-cause inpatient and postdischarge mortality

	Inpatient mortality					One year postdischarge mortality				
	Deaths (n=381)	Univariate model		Multivariable model		Mortality rate/1000 CYs (95% CI)	Univariate model		Multivariable model	
		SHR (95% CI)	P value	aSHR (95% CI)*	P value		HR (95% CI)	P value	aHR (95% CI)*	P value
Anaemia status										
None	47 (2.6)	Reference		Reference		25.8 (18.6‒35.7)	Reference		Reference	
Mild	59 (2.9)	1.11 (0.92‒1.34)	0.29	1.05 (0.92‒1.19)	0.47	19.4 (13.6‒27.6)	0.72 (0.42‒1.24)	0.24	0.60 (0.35‒1.03)	0.07
Moderate	170 (4.4)	1.70 (1.48‒1.96)	<0.001	1.38 (1.12‒1.69)	0.002	36.9 (30.4‒44.6)	1.50 (0.97‒2.31)	0.07	1.06 (0.69‒1.62)	0.80
Severe	88 (7.7)	3.07 (2.66‒3.55)	<0.001	1.94 (1.71‒2.20)	<0.001	59.2 (44.2‒79.2)	2.64 (1.53‒4.57)	0.001	1.94 (1.11‒3.40)	0.02
Not available	17 (3.4)	1.31 (1.06‒1.61)	0.01	1.83 (1.52‒2.21)	<0.001	31.6 (17.9‒55.6)	1.26 (0.60‒2.65)	0.54	1.46 (0.63‒3.40)	0.38
Malaria parasitaemia										
None	313 (4.1)	Reference		Reference		37.8 (32.9‒43.3)	Reference		Reference	
Low	15 (4.1)	0.99 (0.87‒1.13)	0.93	1.20 (1.13‒1.27)	<0.001	6.92 (1.73‒27.7)	0.16 (0.04‒0.68)	0.01	0.26 (0.06‒1.10)	0.07
Medium	18 (2.3)	0.56 (0.48‒0.64)	<0.001	0.67 (0.63‒0.72)	<0.001	9.27 (4.17‒20.6)	0.22 (0.09‒0.52)	0.001	0.35 (0.15‒0.84)	0.02
High	14 (6.3)	1.53 (1.26‒1.87)	<0.001	2.37 (2.00‒2.80)	<0.001	11.2 (2.79‒44.7)	0.27 (0.07‒1.19)	0.08	0.53 (0.12‒2.23)	0.38
Not available	21 (4.3)	1.05 (0.87‒1.27)	0.61	2.21 (1.69‒2.90)	<0.001	30.3 (16.8‒54.7)	0.76 (0.38‒1.50)	0.43	1.33 (0.60‒2.94)	0.48

* Adjusted for age, HIV, malnutrition, sickle cell, cerebral palsy, heart disease, known epilepsy, bacteraemia, season, year of admission, KHDSS area and blood transfusion, low malaria parasitaemia; <2500/mL, medium malaria parasitaemia; 2500–250 000/mL, high malaria parasitaemia; >250 000/mL, no anaemia; haemoglobin ≥11g/L, mild anaemia; haemoglobin 10–10.9 g/L, moderate anaemia; haemoglobin 7–9.9 g/L, severe anaemia; haemoglobin <7g/L, CYs; child-years of follow-up, mortality rates are reported with 95% CIs, SHR; subdistribution HRs from competing risk models, HR from multilevel mixed-effects parametric survival regression analysis with Weibull distribution.

aHR, adjusted HR; aSHR, adjusted subdistribution HR; CYs, child-years; KHDSS, Kilifi Health Demographic Surveillance System; SHR, subdistribution HR.

Of the 381 inpatient deaths, 43 (11%) and 20 (5.2%) were assigned malaria and anaemia diagnoses, with lower respiratory tract infection and malnutrition assigned among 78 (20%) and 59 (15%) deaths, respectively. Inpatient deaths among children with malaria (any MPD level) 36/47 (77%) were more likely to be assigned a malaria diagnosis at death than inpatient deaths among children with anaemia (all anaemia levels), where 19/317 (6.0%) were assigned a diagnosis of anaemia at death (p<0.001) (online supplemental table 6). The inpatient deaths for other diagnoses are shown in online supplemental table 7.

### Postdischarge deaths

There were 9050 live discharges, of which 6 (<0.1%) were missing follow-up data and therefore 9044 were followed up in the KHDSS for 6957 child-years (figure 1). Overall, there were 228 (3.1%) postdischarge deaths: mortality rate of 32.8 (95% CI 28.8‒37.3) deaths/1000 child-years. The median (IQR) time to postdischarge death was 44 (11‒116) days. Of the 228 deaths, 72 (32%), 135 (59%) and 180 (79%) occurred within 1, 3 and 6 months, respectively (online supplemental table 8). Of the 228 deaths, 129 (57%) occurred in a health facility (97 at KCH) and 99 (43%) in the community. Assigned diagnosis at time of death among 97 deaths at KCH and the community deaths through verbal autopsy records are shown in online supplemental tables 9 and 10, respectively.

Overall, 36/1772 (2.0%), 31/2010 (1.5%), 104/3722 (2.8%) and 45/1051 (4.3%) postdischarge deaths occurred among admissions with no, mild, moderate and severe anaemia, respectively. Admissions with any level of anaemia constituted 79% (180/228) of all postdischarge deaths (figure 1a). There were 207/7259 (2.9%), 2/350 (0.6%), 6/761 (0.8%) and 2/210 (1.0%) postdischarge deaths among admissions with no, low, medium and high MPD, respectively. Admissions with any level of MPD constituted only 4.4% (10/228) of all postdischarge deaths (table 2, figure 1b and online supplemental table 8).

Severe anaemia was positively associated with postdischarge mortality (adjusted HR, aHR 1.94 (95% CI 1.11‒3.40)) (figure 2a and table 3), while mild and moderate anaemia were not. Among children without malaria parasitaemia, severe anaemia was associated with greater than twofold hazard of death (aHR 2.39 (95% CI 1.34‒4.26)); however, among children with malaria parasitaemia, no level of anaemia was associated with mortality (online supplemental table 11).

Overall, medium MPD was negatively associated with postdischarge mortality compared with no parasitaemia (aHR 0.35 (95% CI 0.15‒0.84)) (figure 2b and table 3). However, high and low MPD were not associated with postdischarge mortality. Full multivariable models showing all the independent variables included are shown in online supplemental tables 12 and 13. The risk and direction of postdischarge death across the anaemia and malaria parasitaemia groups including only single admission corresponds to those among all study participants online supplemental table 5.

### Effect of blood transfusion on postdischarge deaths

Overall, blood transfusion was associated with lower hazard of death (pooled HR 0.09 (95% CI 0.001‒0.35)) (online supplemental figure 5). Blood transfusion was only associated with lower risk of postdischarge deaths among children with severe anaemia compared with children not transfused. However, there was no evidence of effect modification, that is, transfusion did not modify the overall effect of anaemia on postdischarge deaths (likelihood ratio test p value=0.16). There were too few postdischarge deaths to explore effect of blood transfusion on MPD strata. Among those requiring blood transfusion during admission, the risk of postdischarge mortality was not different between those transfused and those who did not receive blood (aHR 0.53 (95% CI 0.23‒1.21)).

## Discussion

Among children admitted in a Kenyan rural hospital and resident in a demographic surveillance system, those admitted with severe anaemia and high malaria parasitaemia were more likely to die during index admission. Mortality risk was persistently high among those admitted with severe anaemia during 1 year after discharge, predominantly among children admitted without malaria parasitaemia. Mild and moderate anaemia were not associated with postdischarge mortality. Following discharge from hospital, deaths among children admitted with high malaria parasitaemia were rare.

Most (59%) postdischarge deaths occurred within 3 months, highlighting vulnerability in this period and the need to carefully plan the transition from inpatient to community care. It was noticeable that only 57% of all postdischarge deaths occurred in a health facility (during a readmission). While it is possible that some of these postdischarge deaths could be averted by better inpatient care or earlier representation, caregivers may delay seeking care because of economic, personal, household-level and health systems constraints.^29^

Our findings are consistent with recent systematic reviews.^4 11^ Children discharged after severe anaemia are likely to experience impaired haematological recovery, even following blood transfusion.^30 31^ This might also increase risks of recurrent severe anaemia, infections and rehospitalisation.^4 5^ In Malawi, Uganda and Kenya, up to 89% of children with severe anaemia were readmitted to hospital with malaria and at least one-third of postdischarge deaths were attributed to malaria.^4 5 8 11^ We found that underlying medical conditions including SM, HIV infection and sickle cell disease were more frequent in severe anaemia admissions. These high-risk children admitted with severe anaemia were linked with respective outpatient care for the underlying medical conditions. However, because of healthcare access and household economical constraints, it is likely most of these children did not attend these outpatient clinics. Data on access and compliance to treatment after hospital discharge were not available. Children with severe anaemia and blood transfused had lower risk of postdischarge mortality potentially because those who survive to transfusion in hospital had better long-term prognosis, reinforcing the need for availability of transfusion services in such settings.^17^

The low postdischarge mortality among children with high malaria parasitaemia is not an isolated finding.^4 11^ Previously, only 0.7% and 1.3% children with severe malaria died in Kenya and Uganda during 6 months postdischarge, respectively.^5 14^ This is also consistent with the declining incidence of malaria and case fatality in coastal Kenya among children <5 years, with more severe malaria being observed among older children above 5 years old.^16 21^ It is also plausible that high malaria parasitaemia will be treated quickly by admitting clinician and has a better prognosis while for children with severe anaemia and underlying undernutrition, HIV and sickle cell diseases will not have resolved by time of discharge. For inpatient mortality, a U-shaped relationship may be because children with low MPD had other severe illnesses with higher case fatality than malaria such as severe pneumonia or sepsis.^13 32^ Despite this finding, malaria may contribute to recurrent severe anaemia, slow haematological recovery and to non-fatal morbidity after hospital discharge.^33 34^

Research has mostly focused on improving inpatient paediatric outcomes, but recent findings including our results show the need to invest more in postdischarge period. WHO updated the malaria chemoprevention guidelines in 2022 to include postdischarge malaria chemoprevention for children admitted to hospital with severe anaemia in moderate to high malaria transmission areas.^35^ However, recent work in sub-Saharan Africa and South Asia suggests that pathways to postdischarge mortality are complex with diverse risks and therefore effective interventions to improve survival should cut across many domains of vulnerability and risk.^6 34^ Children generally return to the same home environment; hence, these exposures continue along with challenges of accessing healthcare after discharge.^30^

The main strength of this study was its large number of hospital admissions spanning one decade and very low attrition (<1%). However, the study only included admissions in a single hospital and residents within KHDSS, thus limiting generalisability across different settings including locally outside the KHDSS. Approximately 5% of the participants were missing data on anaemia and malaria parasitaemia diagnosis which were included as ‘not available’ category, a potential source for misclassification bias. The hospital data are limited in its ability to distinguish primary diagnosis and comorbidity; therefore, we are limited in estimating the true burden and effect of either anaemia or malaria parasitaemia on mortality. As a surveillance system, it lacked systematic, comprehensive data on household, caregiver, social–economical, prior healthcare access and compliance with treatment after discharge. There was no systematic genotyping for sickle cell disease, it is likely we under-reported cases of this disease. Causes of postdischarge deaths occurring outside KCH were estimated through verbal autopsy, with inherent limitations.

## Conclusions

Children admitted with severe anaemia are at high risk of death 1 year following hospital discharge. However, postdischarge deaths were rare following admission with malaria parasitaemia. Testing and implementing interventions targeting high risk children for postdischarge care among children with severe anaemia which address the complex background vulnerabilities may provide opportunities to improve long-term survival in this population.

## Supplementary material

10.1136/bmjgh-2024-016600 online supplemental file 1

## Data Availability

Data are available in a public, open access repository.
