# Supplementary material for: Inpatient and postdischarge mortality among children with anaemia and malaria parasitaemia in Kenya: a cohort study
Source: BMJ Glob Health. 2024 Oct 21;9(10):e016600. doi: 10.1136/bmjgh-2024-016600 (PMC11499763; doi:10.1136/bmjgh-2024-016600)
Supplement: online supplemental file 1 [file bmjgh-9-10-s001.pdf]

Inpatient and post-discharge mortality among children with anaemia and malaria parasitaemia in Kenya: a cohort study.

Online supplement material

Table of Contents

Supplement methods....4

Study setting. 4

Supplement Figure 1. Map of Kilifi Health and Demographic Surveillance System (KHDSS) showing the three regions (North, South of creek and the Kilifi Township). 5

Data sources and clinical definition. 5

Verbal autopsy. 6

Selection of Parametric survival regression models. 7

Supplement Table1. Selection of best fitting probability distribution using information criterion tests. 7

Supplement Figure 2. Coxsnell residual plots for selecting best fitting probability distribution for Malaria Parasitaemia model: a) Gompertz, b) Weibull, c) Lognormal and d) Exponential distribution. . 8

Supplement Figure 3. Coxsnell residual plots for selecting best fitting probability distribution for Anaemia model: a) Gompertz, b) Weibull, c) Lognormal and d) Exponential distribution. 9

Supplementary Results 10

Supplement Table 2a. Child characteristics at admission by anaemia level and malaria parasitaemia density....10

Supplement Table 2b. Admission characteristics by Malaria Parasitaemia and Anaemia categories among non-KHDSS residents. 13

Supplement Table 3. Admission characteristics by KHDSS resident....15

Supplement Table 4. Inpatient case fatality and one-year post-discharge mortality rates among children admitted at KCH and resident within KHDSS....17

Supplement Table 5. Anaemia levels and malaria parasitaemia density and all-cause inpatient and post-discharge mortality including single admissions only. 18

Supplement Table 6. Diagnosis assigned at death (all inpatient deaths). 19

Supplement Table 7. Leading diagnoses assigned at discharge and their case fatalities....20

Supplement Table 8. Timing of post-discharge deaths among children admitted with anaemia and Malaria parasitaemia. 20

Supplement Table 9. Diagnosis assigned at death (post-discharge deaths at KCH). 21

Supplement Table 10. Diagnosis assigned at death (post-discharge deaths outside KCH assigned through verbal autopsy in the community)....22

Supplement Table 11. The effects of anaemia levels on post-discharge mortality among children with and without a comorbidity of and level of malaria parasitaemia....23

Supplement Table 12. The effect of admission with various levels of anaemia on inpatient deaths and all-cause post-discharge mortality (full multivariable models). 24

Supplement Table 13. The effect of admission with Malaria Parasitaemia on inpatient deaths and all-cause post-discharge mortality (full multivariable models). 25

Supplementary Figures 26
Supplement Figure 4. Stacked bar chart of a) Malaria parasitaemia and b) Anaemia by age groups (age in months). 26
Supplement Figure 5. Effect of blood transfusion on risk of post-discharge deaths among admissions with anaemia. 27
STROBE Statement 28
Author Reflexivity Statement 31
References 34

Supplement methods

Study setting.

The study was conducted among children admitted at Kilifi County Hospital (KCH) who are residents of the Kilifi Health and Demographic Surveillance System (KHDSS). KCH is a level IV hospital offering both outpatient and inpatient care with two paediatric wards; a general ward with 70 beds and a high dependency unit (HDU) with 15 beds managed by research nurses and clinicians. KCH serves approximately 5,000 paediatric admissions annually.[1-3] KHDSS was established in 2002 to collect community-based data on vital status (births, migration, pregnancies and deaths) among approximately 260,000 people who reside within 891km^2 south and north of Kilifi creek. The population is enumerated every four months. At the centre of KHDSS is the KCH and creek extending from the Indian Ocean. Around the creek is the urban Kilifi town coded as Kilifi Township. The creek divides KHDSS into two (apart from Kilifi Township), the south and north of creek as shown in **online supplemental figure 1** below. KHDSS covers 40 sub-location administrative units, 24 in the south, 3 in Kilifi Township and 13 in the North. The south of the Kilifi creek continues to have moderate malaria transmission while north of creek has experienced remarkable reduction in parasite prevalence (to levels <1%).[4,5] The population is enumerated every four months, and these data are linked to the KCH admissions through unique identifiers. Malaria transmission. Children admitted to KCH, but resident outside the KHDSS, were excluded because they lacked follow-up data after hospital discharge.

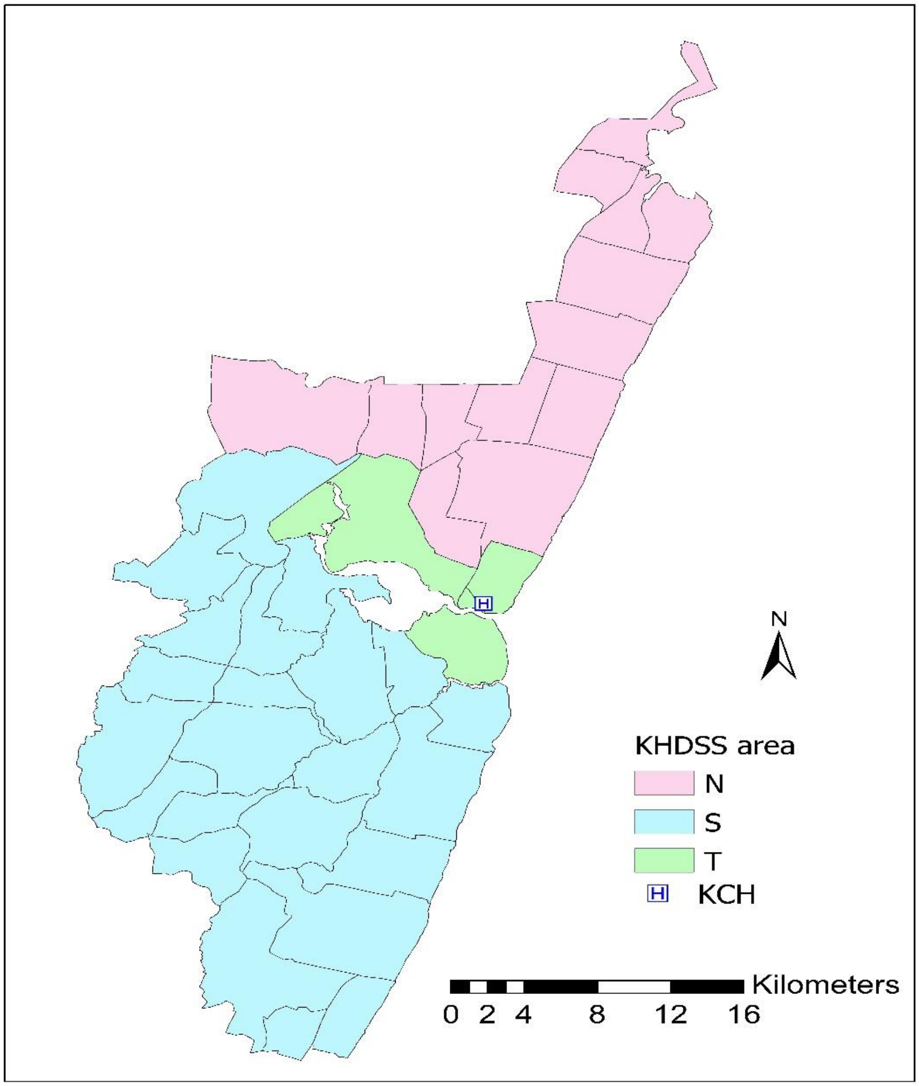

Supplement Figure 1. Map of Kilifi Health and Demographic Surveillance System (KHDSS) showing the three regions (North, South of creek and the Kilifi Township).

N; North of creek, S; South of creek, T; Kilifi Township, KCH; Kilifi County Hospital, the white region between south of creek and Kilifi Township is the creek extending from Indian Ocean.

Data sources and clinical definition.

The complete blood cell count was performed using a DxH 520 haematology analyser (Beckman Coulter, CA, USA). From 2007, HIV testing using Determine (Inverness Medical, FL, USA) and Unigold (Trinity Biotech, Bray, Ireland) was offered to all paediatric admissions. Giemsa-stained thick and thin blood films were examined for *Plasmodium falciparum* parasites according to

standard methods and data were presented as malaria parasite density (MPD), i.e. the number of infected red blood cells (RBCs) per 500, 200 or 100 RBC total counted. Parasitaemia per µl of blood was calculated as (the number of parasitised RBCs x number of RBCs per µl) / (number of RBCs counted or number of parasites counted times the number of white blood cells (WBCs) per µl/number of WBCs counted. Where data on actual number of RBCs or number of WBCs per µl of blood was not available, 5 million RBC and 8,000 WBC per µl of blood were assumed.

Childrens' weights (to the nearest 0.1 kg) were measured using electronic scale (Seca, Birmingham, UK), Mid-upper arm circumference (MUAC) using non-stretch measuring tape (TALC, St Albans, UK) and length (to the nearest 0.1 cm) for children younger than 2 years using a standard UNICEF measuring board or height measured using wall-mounted stadiometer (Seca, Birmingham, UK).

Hypoxia was defined as oxygen saturation <90% measured using pulse oximetry (at sea level). Severe pneumonia was defined using clinical signs following the WHO 2013 guidelines as presence of difficulty breathing or cough plus any of the following signs impaired consciousness, central cyanosis, inability to breastfeed or drink or vomiting everything, lower chest wall indrawing or hypoxia.[6]

We report wasting defined using the middle upper arm circumference (MUAC) because MUAC is less affected by dehydration compared to weight-based anthropometry,[7] easier to measure than length-based indices in sick children, and had the least missing records.

Verbal autopsy.

Since January 2008, causes of deaths occurring in the community within the KHDSS have been assigned using verbal autopsy (VA). Data on community deaths are collected using the World Health Organization (WHO) Sample Vital Registration with Verbal Autopsy (SAVVY) tools.

Medical doctors trained how to use the WHO International Classification of Diseases 10th revision (ICD-10) reviews the WHO SAVVY VA questionnaires and determines the immediate and underlying causes of death.[8]

Selection of Parametric survival regression models.

The best fitting probability distribution was selected based on two information criterion tests: a) Akaike Information Criterion (AIC) and Bayesian Information Criterion (BIC) and visual inspection of the cox-snell residuals plots. The distribution that minimized loss of information, i.e the one with the least AIC and BIC was considered the best fitting. Using the cox-snell residuals plots, the distribution closest to the hypothesized straight line through the origin was considered most fitting. Four common parametric survival distributions were assessed: a) Exponential, Lognormal, Gompertz and Weibull. The results of information criterion tests are shown in the table below. Weibull distribution was selected because it had the lowest AIC and BIC (**online supplemental table 1**).

Supplement Table1. Selection of best fitting probability distribution using information criterion tests.

	Malaria parasitaemia Model			Anaemia Model		
Survival Distribution	AIC	BIC	Best fitting distribution selected	AIC	BIC	Best fitting distribution selected
Gompertz	2762.139	2811.429	No	2766.055	2815.345	No
Weibull	2756.831	2806.121	Yes	2760.645	2809.935	Yes
Lognormal	2761.124	2810.415	No	2765.043	2814.333	No
Exponential	2883.55	2925.798	No	2887.712	2929.96	No

AIC; Akaike Information Criterion, BIC; Bayesian Information Criterion, Weibull distribution had both the lowest AIC and BIC.

Supplement Figures 2 and 3 show the Weibull distribution was the best fitting model to our data.

Supplement Figure 2. Coxsnell residual plots for selecting best fitting probability distribution for Malaria Parasitaemia model: a) Gompertz, b) Weibull, c) Lognormal and d) Exponential distribution.

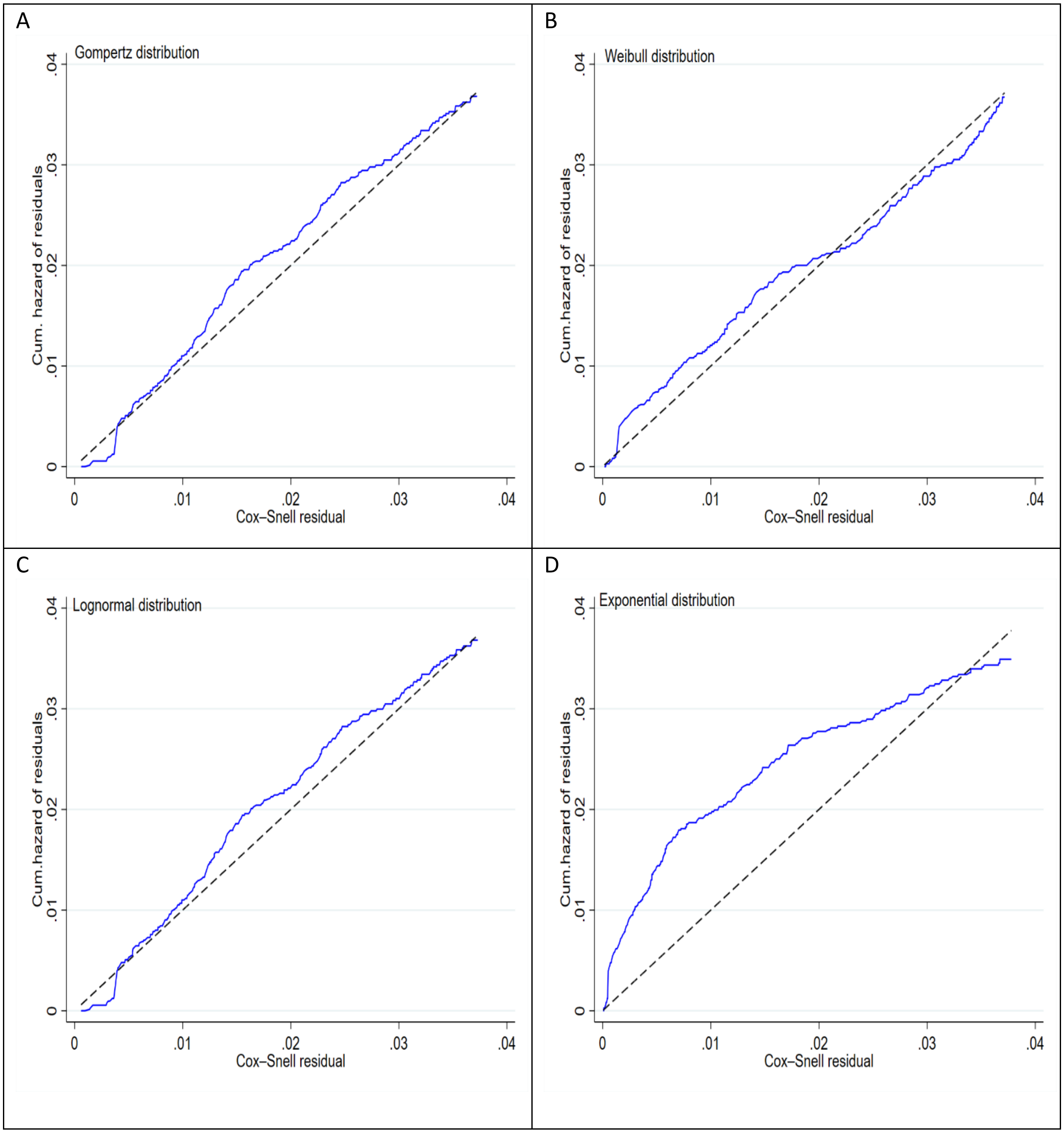

Supplement Figure 3. Coxsnell residual plots for selecting best fitting probability distribution for Anaemia model: a) Gompertz, b) Weibull, c) Lognormal and d) Exponential distribution.

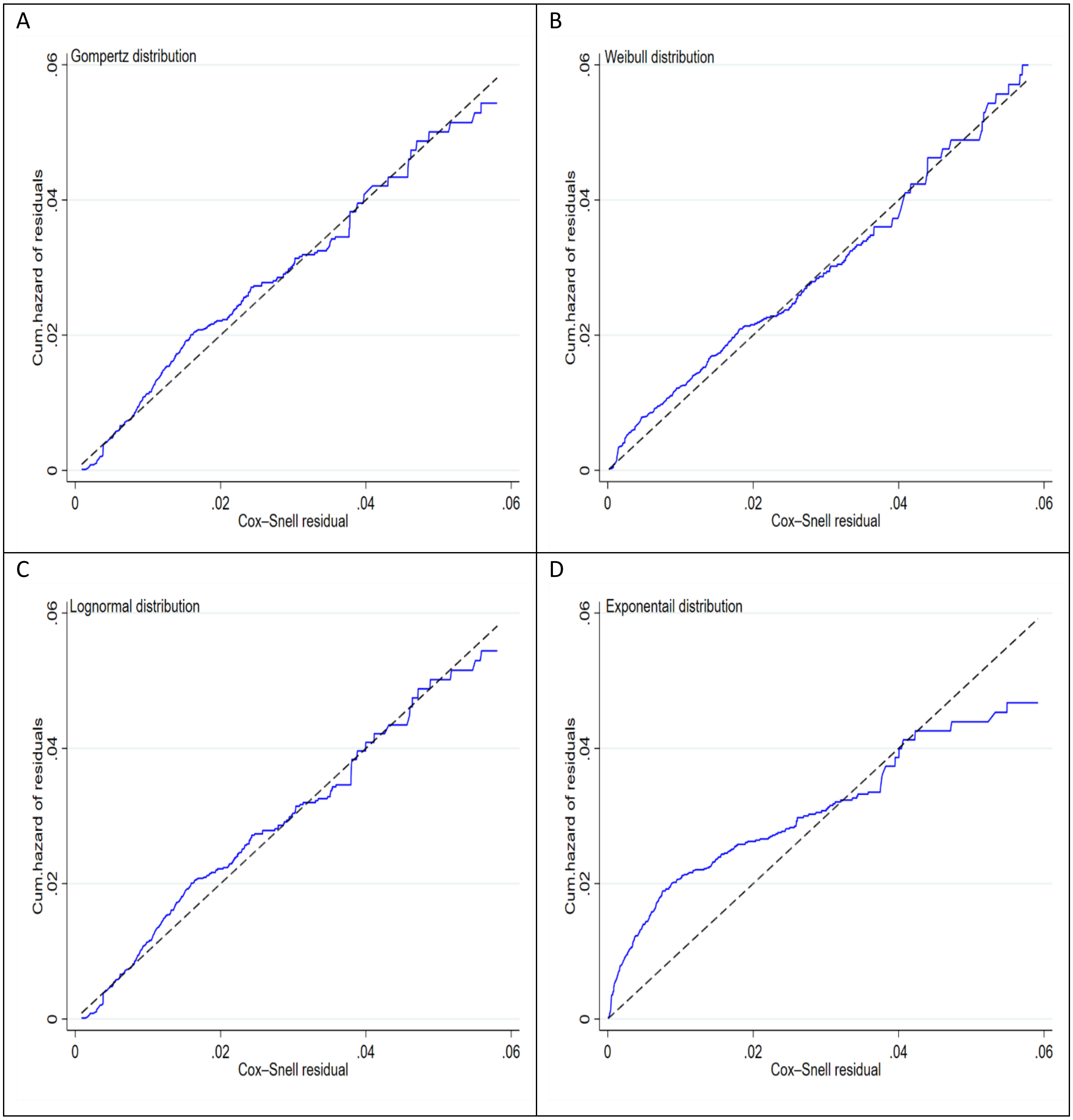

Supplementary Results

Supplement Table 2a. Child characteristics at admission by anaemia level and malaria parasitaemia density.

**Characteristics**	**Anaemia**					**Malaria Parasitaemia**					**All (n=9431)**
	None (n=1822)	Mild (n=2069)	Moderate (n=3893)	Severe (n=1140)	Not available (n=507)	None (n=7576)	Low (n=366)	Medium (n=779)	High (n=224)	Not available (n=486)	
Sex –Female	817 (45)	890 (43)	1627 (42)	507 (44)	203 (40)	3223 (43)	171 (47)	344 (44)	103 (46)	203 (41)	4044 (43)
Age in months											
<6	171 (9.4)	307 (15)	615 (16)	56 (4.9)	82 (16)	1133 (15)	5 (1.4)	13 (1.7)	1 (0.5)	79 (16)	1231 (13)
6–11	205 (11)	341 (16)	838 (22)	204 (18)	96 (19)	1490 (20)	21 (5.7)	59 (7.6)	15 (6.7)	99 (20)	1684 (18)
12–23	430 (24)	574 (28)	1196 (31)	312 (27)	166 (33)	2236 (30)	72 (20)	171 (22)	50 (22)	149 (31)	2678 (28)
≥24	1016 (56)	847 (41)	1244 (32)	568 (50)	163 (32)	2717 (36)	268 (73)	536 (69)	158 (71)	159 (33)	3838 (41)
Median days of hospital stay [IQR]											
Survivors	3 (2–5)	3 (2–5)	3 (2–6)	4 (3–7)	1 (0–3)	3 (2–6)	3 (2–5)	3 (2–4)	3 (2–4)	1 (0–3)	3 (2–6)
Deaths	3 (1–8)	3 (1–8)	2 (1–8)	1 (0–3)	0 (0–1)	2 (1–7)	2 (1–3)	1 (0–1)	1 (0–1)	0 (0–1)	2 (1–6)
Received blood transfusion	20 (1.1)	19 (0.9)	61 (1.6)	384 (34)	4 (0.8)	339 (4.5)	41 (11)	84 (11)	12 (5.4)	12 (2.5)	488 (5.2)
Repeated admissions	284 (16)	364 (18)	787 (20)	246 (22)	101 (20)	1536 (20)	37 (10)	95 (12)	28 (13)	86 (18)	1782 (19)
KHDSS region											
North	2516 (33)	28 (7.7)	64 (8.2)	12 (5.4)	132 (27)	2516 (33)	28 (7.7)	64 (8.2)	12 (5.4)	132 (27)	2752 (29)
Kilifi Township	2101 (28)	35 (9.6)	76 (9.8)	23 (10)	174 (36)	2101 (28)	35 (9.6)	76 (9.8)	23 (10)	174 (36)	2409 (26)
South	2959 (39)	303 (83)	639 (82)	189 (84)	180 (37)	2959 (39)	303 (83)	639 (82)	189 (84)	180 (37)	4270 (45)
Season of the year											
Dry	899 (49)	1005 (49)	2011 (52)	584 (51)	287 (57)	3788 (50)	201 (55)	406 (52)	110 (49)	281 (58)	4786 (51)
Rainy	923 (51)	1064 (51)	1882 (48)	556 (49)	220 (43)	3788 (50)	165 (45)	373 (48)	114 (51)	205 (42)	4645 (49)
**Nutritional status**											
Not wasted	1589 (87)	1745 (84)	3061 (79)	823 (72)	435 (86)	5991 (79)	340 (93)	710 (91)	208 (93)	404 (83)	7653 (81)
Moderate wasted	111 (6.1)	163 (7.9)	409 (11)	153 (13)	38 (7.5)	777 (10)	11 (3.0)	31 (3.9)	11 (4.9)	44 (9.1)	874 (9.3)
Severe wasted	108 (5.9)	146 (7.1)	405 (10)	158 (14)	25 (4.9)	770 (10)	12 (3.3)	30 (3.9)	3 (1.3)	27 (5.6)	842 (8.9)
Missing MUAC	14 (0.8)	15 (0.7)	18 (0.5)	6 (0.5)	9 (1.8)	38 (0.5)	3 (0.8)	8 (1.0)	2 (0.9)	11 (2.3)	62 (0.7)
Kwashiorkor	26 (1.4)	40 (1.9)	167 (4.3)	85 (7.5)	4 (0.8)	276 (3.6)	12 (3.3)	24 (3.1)	3 (1.3)	7 (1.4)	322 (3.4)
**Clinical signs**											
Axillary temperature											
<36°C	128 (7.0)	127 (6.1)	199 (5.1)	46 (4.0)	29 (5.7)	453 (5·9)	24 (6.6)	18 (2.3)	10 (4.5)	24 (4.9)	529 (5.6)
36 to 37·5°C	864 (47)	904 (44)	1547 (40)	453 (40)	273 (54)	3428 (45)	104 (28)	198 (25)	48 (21)	263 (54)	4041 (43)
>37·5 to 39°C	579 (32)	746 (36)	1494 (38)	485 (43)	159 (31)	2743 (36)	150 (41)	322 (41)	94 (42)	154 (32)	3463 (37)
>39°C	249 (14)	291 (14)	652 (17)	156 (14)	42 (8.3)	949 (13)	87 (24)	241 (31)	72 (32)	41 (8.4)	1390 (15)
Missing	2 (0.1)	1 (0.05)	1 (0.03)	0	4 (0.8)	3 (0.04)	1 (0.3)	0	0	4 (0.8)	8 (0.08)
Respiratory rate											
Bradypnoea	75 (4.1)	98 (4.7)	133 (3.4)	34 (2.9)	37 (7.3)	305 (4.0)	8 (2.2)	26 (3.3)	3 (1.3)	35 (7.2)	377 (4.0)

Normal	1177 (65)	1310 (63)	2441 (63)	640 (56)	344 (68)	4814 (64)	206 (56)	441 (57)	131 (58)	320 (66)	5912 (63)
Tachypnoea	568 (31)	655 (32)	1306 (34)	464 (41)	97 (19)	2436 (32)	152 (42)	310 (40)	90 (40)	102 (21)	3090 (33)
Missing	2 (0.1)	6 (0.3)	13 (0.3)	2 (0.2)	29 (5.7)	21 (0.3)	0	2 (0.3)	0	29 (6.0)	52 (0.6)
Heart rate											
Bradycardia	30 (1.7)	27 (1.3)	70 (1.8)	25 (2.2)	14 (2.8)	139 (1.8)	7 (1.9)	10 (1.3)	2 (0.9)	8 (1.7)	166 (1.8)
Normal	894 (49)	986 (48)	1819 (47)	445 (39)	275 (54)	3689 (49)	143 (39)	250 (32)	74 (33)	263 (54)	4419 (47)
Tachycardia	898 (49)	1055 (51)	2003 (51)	670 (59)	216 (43)	3746 (49)	216 (59)	519 (67)	148 (66)	213 (44)	4842 (51)
Missing	0	1 (0.05)	1 (0.03)	0	2 (0.4)	2 (0.03)	0	0	0	2 (0.4)	4 (0.04)
Hypoxia	92 (9.1)	96 (4.6)	293 (7.5)	76 (6.7)	20 (3.9)	515 (6.8)	11 (3.0)	27 (3.5)	6 (2.7)	18 (3.7)	577 (6.1)
Breathing difficulty	474 (26)	649 (31)	1476 (38)	364 (32)	112 (22)	2763 (36)	55 (15)	117 (15)	29 (13)	111 (23)	3075 (33)
Lower chest indrawing	421 (23)	577 (28)	1342 (34)	266 (23)	79 (16)	2515 (33)	27 (7.4)	54 (6.9)	13 (5.8)	76 (16)	2685 (28)
Wheeze	128 (7.0)	130 (6.3)	222 (5.7)	15 (1.3)	26 (5.1)	491 (6.5)	2 (0.6)	5 (0.6)	0	23 (4.7)	521 (5.5)
**Signs of shock and dehydration**											
Capillary refill >2s	37 (2.0)	31 (1.5)	69 (1.8)	90 (7.9)	11 (2.2)	180 (2.4)	11 (3.0)	22 (2.8)	13 (5.8)	12 (2.5)	238 (2.5)
Weak pulse	39 (2.1)	31 (1.5)	58 (1.5)	56 (4.9)	10 (1.9)	156 (2.1)	8 (2.2)	12 (1.5)	7 (3.1)	11 (2.3)	194 (2.1)
Sunken eyes	250 (14)	244 (12)	403 (10)	72 (6.3)	43 (8.5)	929 (12)	14 (3.8)	21 (2.7)	11 (4.9)	37 (7.6)	1012 (11)
Reduced skin turgor	135 (7.4)	112 (5.4)	167 (4.3)	43 (3.8)	16 (3.2)	439 (5.8)	3 (0.8)	4 (0.5)	7 (3.1)	20 (4.1)	473 (5.0)
No dehydration	1568 (86)	1801 (87)	3388 (87)	1063 (93)	463 (91)	6512 (86)	356 (97)	758 (97)	215 (96)	442 (91)	8283 (88)
Some dehydration	102 (5.6)	112 (5.4)	220 (5.7)	32 (2.8)	22 (4.3)	451 (6.0)	5 (1.4)	13 (1.7)	2 (0.9)	17 (3.5)	488 (5.2)
Severe dehydration	152 (8.3)	156 (7.5)	285 (7.3)	45 (4.0)	22 (4.3)	613 (8.1)	5 (1.4)	8 (1.0)	7 (3.1)	27 (5.6)	660 (7.0)
Impaired consciousness	147 (8.1)	197 (9.5)	414 (11)	216 (19)	23 (4.5)	618 (8.2)	108 (30)	194 (25)	53 (24)	24 (4.9)	997 (11)
Convulsion	501 (28)	634 (31)	868 (22)	176 (15)	68 (13)	1362 (18)	206 (56)	492 (63)	138 (62)	49 (10)	2247 (24)
Pallor	141 (7.7)	193 (9.3)	699 (18)	819 (72)	30 (5.9)	1334 (18)	143 (39)	278 (36)	84 (38)	43 (8.9)	1882 (20)
**Diagnosis/laboratory features**											
Severe pneumonia	448 (25)	618 (30)	1435 (37)	335 (29)	88 (17)	2640 (35)	64 (17)	107 (14)	27 (12)	86 (18)	2924 (31)
Diarrhoea	407 (22)	435 (21)	777 (20)	152 (13)	85 (17)	1668 (22)	27 (7.4)	55 (7.1)	21 (9.4)	85 (17)	1856 (20)
HIV status	25 (1.4)	43 (2.1)	151 (3.9)	40 (3.5)	8 (1.6)	249 (3.3)	2 (0.6)	4 (0.5)	3 (1.3)	9 (1.9)	267 (2.8)
Bacteraemia	55 (3.0)	68 (3.3)	217 (5.6)	92 (8.1)	4 (0.8)	399 (5.3)	11 (3.0)	19 (2.4)	3 (1.3)	4 (0.8)	436 (4.6)
Epilepsy	64 (3.5)	88 (4.3)	92 (2.4)	10 (0.9)	13 (2.6)	250 (3.3)	5 (1.4)	2 (0.3)	1 (0.5)	9 (1.9)	267 (2.8)
Heart disease	40 (2.2)	32 (1.6)	87 (2.2)	7 (0.6)	8 (1.6)	162 (2.1)	1 (0.3)	1 (0.1)	1 (0.5)	9 (1.9)	174 (1.8)
Sickle cell disease	4 (0.2)	3 (0.1)	199 (5.1)	200 (18)	8 (1.6)	398 (5.3)	1 (0.3)	2 (0.3)	0	13 (2.7)	414 (4.4)
Cerebral palsy	24 (1.3)	16 (0.8)	40 (1.0)	9 (0.8)	3 (0.6)	86 (1.1)	3 (0.8)	0	0	3 (0.6)	92 (1.0)
Anaemia											
None	-	-	-	-	-	1618 (21)	51 (14)	111 (14)	28 (13)	14 (2.9)	1822 (19)
Mild	-	-	-	-	-	1788 (24)	59 (16)	150 (19)	52 (23)	20 (4.1)	2069 (22)
Moderate	-	-	-	-	-	3306 (44)	155 (42)	284 (36)	104 (46)	44 (9.1)	3893 (41)
Severe	-	-	-	-	-	759 (10)	100 (27)	228 (29)	39 (17)	14 (2.9)	1140 (12)
Not available	-	-	-	-	-	105 (1.4)	1 (0.3)	6 (0.8)	1 (0.5)	394 (81)	507 (5.4)
Malaria parasitaemia											
None	1618 (89)	1788 (86)	3306 (85)	759 (67)	105 (21)	-	-	-	-	-	7576 (80)
Low	51 (2.8)	59 (2.9)	155 (4.0)	100 (8.8)	1 (0.2)	-	-	-	-	-	366 (3.9)
Medium	111 (6.1)	150 (7.3)	284 (7.3)	228 (20)	6 (1.2)	-	-	-	-	-	779 (8.3)
High	28 (1.5)	52 (2.5)	104 (2.7)	39 (3.4)	1 (0.2)	-	-	-	-	-	224 (2.4)

Not available	14 (0.8)	20 (1.0)	44 (1.1)	14 (1.2)	394 (78)	-	-	-	-	-	486 (5.2)

KHDSS; Kilifi Health Demographic Surveillance System, Low malaria parasitaemia; <2500/µl, medium malaria parasitaemia; 2500 to 250000/µl, High malaria parasitaemia; >250000/µl, no anaemia; haemoglobin ≥11g/dl, mild anaemia; haemoglobin 10 to 10.9 g/dl, moderate anaemia; haemoglobin 7 to 9.9 g/dl, severe anaemia; haemoglobin <7g/dl, bradypnoea; respiratory rate<30/min for those aged <12, <24 for those aged 12 to <36 and <22 for those aged ≥36 months, normal respiratory rate; respiratory rate 30 to 60 for those aged <12, 24 to 40 for those aged 12 to <36 and 22 to 34 for those aged ≥36 months, tachypnoea; respiratory rate >60 for those aged <12, >40 for those aged 12 to <36 and >34 for those aged ≥36 months, bradycardia; heart rate<100/min for those aged <12, <90 for those aged 12 to <36 and <80 for those aged ≥36 months, normal heart rate; heart rate 100 to 160 for those aged <12, 90 to 150 for those aged 12 to <36 and 80 to 140 for those aged ≥36 months, tachycardia; heart rate >160 for those aged <12, >150 for those aged 12 to <36 and >140 for those aged ≥36 months, rainy season is from April to June and October to December.

Supplement Table 2b. Admission characteristics by Malaria Parasitaemia and Anaemia categories among non-KHDSS residents.

**Characteristics**	**Malaria Parasitaemia**					**Anaemia**					**All (n=9482)**
	None (n=7980)	Low (n=338)	Medium (n=572)	High (n=153)	Not available (n=439)	None (n=1708)	Mild (n=1993)	Moderate (n=4129)	Severe (n=1254)	Not available (n=398)	
Sex –Female	3288 (41)	153 (45)	267 (47)	68 (44)	179 (41)	738 (43)	837 (42)	1660 (40)	556 (44)	164 (41)	3955 (42)
Age in months; Median [IQR]	15 (7.9–28)	32 (21–45)	31 (19–44)	32 (21–43)	16 (8.5–27)	25 (11–40)	16 (7.7–28)	14 (7.8–25)	22 (10–38)	15 (9.0–27)	17 (8.5–31)
Days of hospital stay [IQR]	4 (2–8)	4 (3–6)	3 (2–5)	2 (2–4)	1 (0–4)	3 (2–7)	3 (2–7)	4 (2–8)	4 (3–8)	1 (0–3)	4 (2–7)
Having multiple admissions	1351 (17)	34 (10)	46 (8.0)	18 (12)	88 (20)	266 (16)	291 (15)	677 (16)	233 (19)	70 (18)	1537 (16)
**Nutritional status**											
Not wasted	5526 (69)	300 (89)	510 (89)	141 (92)	337 (77)	1382 (81)	1485 (75)	2792 (68)	837 (67)	318 (80)	6814 (72)
Moderate wasted	1020 (13)	20 (5.9)	39 (6.8)	5 (3.3)	51 (12)	139 (8.1)	220 (11)	556 (13)	179 (14)	41 (10)	1135 (12)
Severe wasted	1386 (17)	15 (4.4)	20 (3.5)	5 (3.3)	43 (9.8)	175 (10)	275 (14)	760 (18)	227 (18)	32 (8.0)	1469 (15)
Missing MUAC	48 (0.6)	3 (0.9)	3 (0.5)	2 (1.3)	8 (1.8)	12 (0.7)	13 (0.7)	21 (0.5)	11 (0.9)	7 (1.8)	64 (0.7)
Kwashiorkor	489 (6.1)	12 (3.6)	22 (3.9)	1 (0.7)	4 (0.9)	39 (2.3)	74 (3.7)	297 (7.2)	112 (8.9)	6 (1.5)	528 (5.6)
**Clinical signs**											
Axillary temp <36^0C	338 (4.2)	14 (4.1)	26 (4.6)	5 (3.3)	27 (6.2)	99 (5.8)	78 (3.9)	149 (3.6)	61 (4.9)	23 (5.8)	410 (4.3)
Axillary temp 36 to 37.5^0C	3435 (43)	119 (35)	162 (28)	47 (231)	222 (51)	835 (49)	851 (43)	1596 (39)	504 (40)	199 (50)	3985 (42)
Axillary temp >37.5 to 39^0C	3075 (39)	147 (43)	238 (42)	53 (35)	126 (29)	549 (32)	782 (39)	1669 (40)	514 (41)	125 (31)	3639 (38)
Axillary temp >39^0C	1127 (14)	58 (17)	146 (26)	48 (31)	63 (14)	224 (13)	280 (14)	714 (17)	174 (14)	50 (13)	1442 (15)
Missing	5 (0.06)	0	0	0	1 (0.2)	1 (0.06)	2 (0.1)	1 (0.02)	1 (0.08)	1 (0.3)	6 (0.06)
Respiratory rate/Min											
Bradypnoea	369 (4.6)	8 (2.4)	20 (3.5)	4 (2.6)	39 (8.9)	92 (5.4)	80 (4.0)	190 (4.6)	42 (3.4)	36 (9.1)	440 (4.6)
Normal	5208 (65)	183 (54)	316 (55)	73 (48)	293 (67)	1085 (64)	1316 (66)	2699 (65)	708 (56)	265 (67)	6073 (64)
Tachypnoea	2389 (30)	146 (43)	234 (41)	76 (50)	86 (20)	527 (31)	594 (30)	1233 (30)	502 (40)	75 (19)	2931 (31)
Missing	14 (0.2)	1 (0.3)	2 (0.4)	0	21 (4.8)	4 (0.2)	3 (0.2)	7 (0.2)	2 (0.3)	22 (5.5)	38 (0.4)
Heart rate/min											
Bradycardia	186 (2.3)	4 (1.2)	10 (1.8)	0	14 (3.2)	40 (2.3)	44 (2.2)	94 (2.3)	26 (2.1)	10 (2.5)	214 (2.3)
Normal	3849 (48)	135 (40)	178 (31)	43 (28)	228 (52)	870 (51)	973 (49)	1875 (45)	511 (41)	204 (51)	4433 (47)
Tachycardia	3943 (49)	199 (59)	383 (67)	110 (72)	197 (45)	797 (47)	976 (49)	2159 (52)	716 (57)	184 (46)	4832 (51)
Missing	2 (0.03)	0	1 (0.2)	0	0	1 (0.06)	0	1 (0.02)	1 (0.08)	0	3 (0.03)
Hypoxia	613 (7.7)	19 (5.6)	19 (3.3)	3 (2.0)	22 (5.0)	134 (7.9)	119 (6.0)	310 (7.5)	90 (7.2)	23 (5.9)	676 (7.1)
Breathing difficulty	2898 (36)	56 (17)	109 (19)	20 (13)	99 (23)	490 (29)	668 (34)	1500 (36)	431 (34)	93 (23)	3182 (34)
Lower chest indrawing	2586 (32)	37 (11)	61 (11)	10 (6.5)	70 (16)	428 (25)	624 (31)	1323 (32)	320 (26)	69 (17)	2764 (29)
Wheeze	398 (5.0)	0	4 (0.7)	0	16 (3.6)	100 (5.9)	105 (5.3)	175 (4.2)	20 (1.6)	18 (4.5)	418 (4.4)
Stridor	76 (0.9)	0	4 (0.7)	0	6 (1.4)	21 (1.2)	17 (0.9)	39 (0.9)	2 (0.2)	7 (1.8)	86 (0.9)
Cyanosis	57 (0.7)	1 (0.3)	1 (0.2)	0	4 (0.9)	31 (1.8)	12 (0.6)	13 (0.3)	3 (0.2)	4 (1.0)	63 (0.7)
Capillary refill >2s	339 (4.3)	14 (4.1)	20 (3.)	4 (2.6)	15 (3.4)	46 (2.7)	57 (2.9)	166 (4.0)	114 (9.1)	9 (2.3)	392 (4.1)
Weak pulse	285 (3.6)	10 (3.0)	13 (2.3)	2 (1.3)	12 (2.7)	52 (3.0)	58 (2.9)	136 (3,3)	68 (5.4)	8 (2.0)	322 (3.4)
Sunken eyes	1177 (15)	15 (4.4)	20 (3.5)	6 (3.9)	37 (8.4)	250 (15)	269 (14)	592 (14)	115 (9.2)	29 (7.3)	1255 (13)

Reduced skin turgor	735 (9.2)	10 (3.0)	10 (1.8)	1 (0.7)	22 (5.0)	159 (9.3)	164 (8.2)	362 (8.8)	76 (6.1)	17 (4.3)	778 (8.2)
No dehydration	6627 (83)	325 (96)	540 (94)	150 (98)	392 (89)	1441 (84)	1689 (85)	3417 (83)	1123 (90)	364 (91)	8034 (85)
Some dehydration	439 (5.5)	2 (0.6)	16 (2.8)	2 (1.3)	13 (3.0)	80 (4.7)	105 (5.3)	236 (5.7)	41 (3.3)	10 (2.5)	472 (5.0)
Severe dehydration	914 (11)	11 (3.3)	16 (2.8)	1 (0.7)	34 (7.7)	187 (11)	199 (9.9)	476 (12)	90 (7.2)	24 (6.0)	976 (10)
Limb temperature gradient	549 (6.8)	20 (5.9)	33 (5.8)	4 (2.6)	19 (4.3)	111 (6.5)	111 (5.6)	294 (7.1)	89 (7.1)	20 (5.0)	625 (6.6)
Impaired consciousness	865 (11)	127 (38)	166 (29)	42 (27)	19 (4.3)	186 (11)	224 (11)	552 (13)	241 (19)	16 (4.0)	1219 (13)
Convulsion	1357(17)	183 (54)	307 (54)	96 (63)	49 (11)	427 (25)	501 (25)	803 (19)	208 (17)	53 (13)	1992 (21)
Pallor	1728 (22)	165 (49)	257 (45)	51 (33)	60 (14)	164 (9.6)	234 (12)	919 (22)	908 (72)	36 (9.1)	2261 (24)
Transfused	424 (5.3)	65 (19)	90 (16)	15 (9.8)	20 (4.6)	20 (1.2)	20 (1.0)	100 (2.4)	472 (38)	2 (0.5)	614 (6.5)
**Diagnosis/laboratory features**											
Severe pneumonia	2780 (35)	62 (18)	104 (18)	21 (14)	77 (18)	475 (28)	665 (33)	1432 (35)	399 (32)	73 (18)	3044 (32)
Diarrhoea	2119 (27)	35 (10)	64 (11)	16 (10)	86 (20)	408 (24)	490 (25)	1129 (27)	217 (17)	76 (19)	2320 (24)
HIV status	413 (5.2)	7 (2.1)	7 (1.2)	1 (0.7)	5 (1.1)	38 (2.2)	68 (3.4)	252 (6.1)	69 (5.5)	6 (1.5)	433 (4.6)
Bacteraemia	455 (5.7)	11 (3.3)	11 (1.9)	2 (1.3)	7 (1.6)	62 (3.6)	90 (4.5)	222 (5.4)	109 (8.7)	3 (0.8)	486 (5.1)
Epilepsy	373 (4.7)	2 (0.6)	5 (0.9)	1 (0.7)	12 (2.7)	99 (5.8)	123 (6.2)	144 (3.5)	9 (0.7)	18 (4.5)	393 (4.1)
Heart disease	209 (2.6)	0	4 (0.7)	0	12 (2.7)	75 (4.4)	47 (2.4)	76 (1.8)	14 (1.1)	13 (3.3)	225 (2.4)
Sickle cell disease	331 (4.2)	8 (2.4)	7 (1.2)	0	11 (2.5)	3 (0.2)	6 (0.3)	188 (4.6)	156 (12)	4 (1.0)	357 (3.8)
Cerebral palsy	170 (2.1)	0	1 (0.2)	0	6 (1.4)	36 (2.1)	44 (2.2)	81 (2.0)	10 (0.8)	6 (1.5)	177 (1.9)
Anaemia											
None	1563 (20)	32 (9.5)	73 (13)	19 (12)	21 (4.8)	-	-	-	-	-	1708 (18)
Mild	1831 (23)	36 (11)	71 (12)	35 (23)	20 (4.6)	-	-	-	-	-	1993 (21)
Moderate	3674 (46)	134 (40)	208 (36)	62 (41)	51 (12)	-	-	-	-	-	4129 (44)
Severe	835 (10)	135 (40)	218 (38)	35 (23)	31 (7.1)	-	-	-	-	-	1254 (13)
Not available	77 (1.0)	1 (0.3)	2 (0.4)	2 (1.3)	316 (72)	-	-	-	-	-	398 (4.2)
Malaria parasitaemia											
None	-	-	-	-		1563 (92)	1831 (92)	3674 (89)	835 (67)	77 (19)	7980 (84)
Low	-	-	-	-		32 (1.9)	36 (1.8)	134 (3.3)	135 (11)	1 (0.3)	338 (3.6)
Medium	-	-	-	-		73 (4.3)	71 (3.6)	208 (5.0)	218 (17)	2 (0.5)	572 (6.0)
High	-	-	-	-		19 (1.1)	35 (1.8)	62 (1.5)	35 (2.8)	2 (0.5)	153 (1.6)
Not available	-	-	-	-		21 (1.2)	20 (1.0)	51 (1.2)	31 (2.5)	316 (79)	439 (4.6)

Low malaria parasitaemia; <2500/ul, medium malaria parasitaemia; 2500 to 250000/ul, High malaria parasitaemia; >250000/ul, no anaemia; haemoglobin ≥11g/dl, mild anaemia; haemoglobin 10 to 10.9 g/dl, moderate anaemia; haemoglobin 7 to 9.9 g/dl, severe anaemia; haemoglobin <7g/dl.

Supplement Table 3. Admission characteristics by KHDSS resident.

Admission characteristic	Outside KHDSS (N=9482)	Inside KHDSS (N=9431)	P-value
Sex –Female	3955 (42)	4044 (43)	0.10
Age in months; Median [IQR]	16.8 (8.5–31.2)	19.5 (9.9–34.2)	<0.001#
Days of hospital stay [IQR]	4 (2-7)	3 (2-6)	<0.001#
Having multiple admissions	1624 (17)	1869 (20)	<0.001
**Nutritional status**			
Not wasted	6814 (72)	7653 (81)	<0.001
Moderate wasted	1135 (12)	874 (9.3)	
Severe wasted	1469 (15)	842 (8.9)	
Missing MUAC	64 (0.7)	62 (0.7)	
Kwashiorkor	528 (5.6)	322 (3.4)	<0.001
**Clinical signs**			
**Axillary temperature**			
Axillary temp <36^{0}C	410 (4.3)	529 (5.6)	<0.001
Axillary temp 36 to 37.5^{0}C	3985 (42)	4041 (43)	
Axillary temp >37.5 to 39^{0}C	3639 (38)	3463 (37)	
Axillary temp >39^{0}C	1442 (15)	1390 (15)	
Missing	6 (0.06)	8 (0.08)	
Respiratory rate/Min			
Bradypnoea	440 (4.6)	377 (4.0)	0.004
Normal	6073 (64)	5912 (63)	
Tachypnoea	2931 (31)	3090 (33)	
Missing	38 (0.4)	52 (0.6)	
Heart rate/min			
Bradycardia	214 (2.3)	166 (1.8)	0.11
Normal	4433 (47)	4419 (47)	
Tachycardia	4832 (51)	4842 (51)	
Missing	3 (0.03)	4 (0.04)	
Hypoxia	676 (7.1)	577 (6.1)	0.005
Breathing difficulty	3182 (34)	3075 (33)	0.23
Lower chest indrawing	2764 (29)	2685 (28)	0.36
Wheeze	418 (4.4)	521 (5.5)	0.001
Stridor	86 (0.9)	70 (0.7)	0.15
Cyanosis	63 (0.66)	34 (0.36)	0.008
Capillary refill >2s	392 (4.1)	238 (2.5)	<0.001
Weak pulse	322 (3.4)	194 (2.1)	<0.001
Sunken eyes	1255 (13)	1012 (11)	<0.001
Reduced skin turgor	778 (8.2)	473 (5.0)	<0.001
Dehydration status			
No dehydration	8034 (85)	8283 (388)	<0.001
Some dehydration	472 (4.9)	488 (5.2)	
Severe dehydration	976 (10)	660 (7.0)	
Limb temperature gradient	625 (6.6)	479 (5.1)	<0.001
Impaired consciousness	1219 (13)	997 (11)	<0.001
Convulsion	1992 (21)	2247 (24)	<0.001
Pallor	2261 (24)	1882 (20)	<0.001

Transfused	614 (6.5)	488 (5.2)	0.001
**Diagnosis/laboratory features**			
Severe pneumonia	3044 (32)	2924 (31)	0.10
Diarrhoea	2320 (24)	1856 (20)	<0.001
HIV status	433 (4.6)	267 (2.8)	<0.001
Bacteraemia	486 (5.1)	436 (4.6)	0.01
Epilepsy	393 (4.1)	267 (2.8)	<0.001
Heart disease	225 (2.4)	174 (1.8)	0.01
Sickle cell disease	357 (3.8)	414 (4.4)	0.03
Cerebral palsy	177 (1.9)	92 (0.9)	<0.001
Anaemia			
None	1708 (18)	1822 (19)	<0.001
Mild	1993 (21)	2069 (22)	
Moderate	4129 (44)	3893 (41)	
Severe	1254 (13)	1140 (12)	
Not available	398 (4.2)	507 (5.4)	
Malaria parasitaemia			
None	7980 (84)	7576 (80)	<0.001
Low	338 (3.6)	366 (3.9)	
Medium	572 (6.0)	779 (8.3)	
High	153 (1.6)	224 (2.4)	
Not available	439 (4.6)	486 (5.2)	
Discharge outcome			
Alive	8807 (93)	9050 (96)	<0.001
Dead	675 (7.1)	381 (4.1)	

Results are N (%) unless specified, IQR; Interquartile range, all P-values are from chi-square/fishers' exact test unless # which are from Wilcoxon rank-sum test.

Supplement Table 4. Inpatient case fatality and one-year post-discharge mortality rates among children admitted at KCH and resident within KHDSS.

	**Inpatient deaths**	**One-year post-discharge deaths**		
	N (%)	N (%) N=228	Child-years	Mortality rate/1000 CYs (95% CI)
All admissions (N=9431)	381(4.0)	228 (3.1)	6957	32.8 (28.8–37.3)
Anaemia				
None	47 (2.6)	36 (2.5)	1396	25.8 (18.6–35.7)
Mild	59 (2.9)	31 (1.7)	1598	19.4 (13.6–27.6)
Moderate	170 (4.4)	104 (3.5)	2822	36.9 (30.4–44.6)
Severe	88 (7.7)	45 (5.5)	761	59.2 (44.2–79.2)
Not available	17 (3.4)	12 (3.0)	380	31.6 (17.9–55.6)
Malaria parasitaemia				
None	313 (4.1)	207 (3.6)	5479	37.8 (32.9–43.3)
Low	15 (4.1)	2 (0.67)	289	6.92 (1.73–27.7)
Medium	18 (2.3)	6 (0.90)	647	9.27 (4.17–20.6)
High	14 (6.3)	2 (1.1)	179	11.2 (2.79–44.7)
Not available	21 (4.3)	11 (2.9)	363	30.3 (16.8–54.7)

CYs; Child-years of follow-up, Mortality rates are reported with 95% confidence intervals, Low malaria parasitaemia; <2500/ul, medium malaria parasitaemia; 2500 to 250000/ul, High malaria parasitaemia; >250000/ul, no anaemia; haemoglobin ≥11g/dl, mild anaemia; haemoglobin 10 to 10.9 g/dl, moderate anaemia; haemoglobin 7 to 9.9 g/dl, severe anaemia; haemoglobin <7g/dl.

Supplement Table 5. Anaemia levels and malaria parasitaemia density and all-cause inpatient and post-discharge mortality including single admissions only.

	Inpatient mortality					One year post-discharge mortality					
	Deaths (N=288)	Univariate model		Multivariable model		Deaths (N=227)	Mortality rate/1000 CYs (95% CI)	Univariate model		Multivariable model	
		SHR (95% CI)	P-value	aSHR (95% CI)*	P-value			HR (95% CI)	P-value	aHR (95% CI)*	P-value
Anaemia status											
None	36 (2.4)	Reference		Reference		36 (2.5)	25.8 (18.6–35.8)	Reference		Reference	
Mild	41 (2.4)	1.01 (0.64–1.57)	0.98	0.96 (0.62–1.50)	0.86	36 (2.2)	22.6 (16.3–31.3)	0.72 (0.42–1.24)	0.24	0.75 (0.47–1.20)	0.24
Moderate	130 (4.2)	1.75 (1.21–2.53)	0.003	1.42 (0.99–2.05)	0.06	102 (3.4)	36.2 (29.8–44.0)	1.50 (0.97–2.31)	0.07	1.00 (0.68–1.47)	0.99
Severe	66 (7.4)	3.18 (2.13–4.76)	<0.001	1.95 (1.22–3.12)	0.005	43 (5.2)	56.1 (41.6–75.7)	2.64 (1.53–4.57)	0.001	1.70 (1.02–2.82)	0.04
Not available	15 (3.6)	1.49 (0.82–2.72)	0.19	2.09 (0.99–4.38)	0.05	10 (2.5)	26.1 (14.1–48.6)	1.26 (0.60–2.65)	0.54	0.89 (0.39–2.03)	0.79
Malaria parasitaemia											
None	226 (3.7)	Reference		Reference		208 (3.6)	38.0 (33.2–43.6)	Reference		Reference	
Low	13 (4.2)	1.11 (0.64–1.94)	0.70	1.22 (0.67–2.21)	0.52	2 (0.67)	6.84 (1.71–27.3)	0.18 (0.05–0.73)	0.01	0.28 (0.07–1.17)	0.08
Medium	17 (2.5)	0.67 (0.41–1.09)	0.11	0.75 (0.45–1.26)	0.28	4 (0.60)	6.21 (2.33–16.6)	0.17 (0.06–0.44)	0.001	0.26 (0.09–0.70)	0.008
High	14 (7.1)	1.93 (1.13–3.30)	0.02	2.69 (1.52–4.74)	0.001	2 (1.09)	11.1 (2.77–44.3)	0.29 (0.07–1.19)	0.09	0.53 (0.13–2.17)	0.38
Not available	18 (4.4)	1.18 (0.73–1.91)	0.49	2.44 (1.30–4.56)	0.005	11 (2.8)	30.0 (16.6–54.1)	0.77 (0.43–1.44)	0.43	1.21 (0.57–2.54)	0.62

*adjusted for age, HIV, malnutrition, sickle cell, cerebral palsy, heart disease, known epilepsy, bacteraemia, season, year of admission, KHDSS area and blood transfusion, Low malaria parasitaemia; <2500/μl, medium malaria parasitaemia; 2500 to 250000/μl, High malaria parasitaemia; >250000/μl, no anaemia; haemoglobin ≥11g/dl, mild anaemia; haemoglobin 10 to 10.9 g/dl, moderate anaemia; haemoglobin 7 to 9.9 g/dl, severe anaemia; haemoglobin <7g/dl, CYs; Child-years of follow-up, Mortality rates are reported with 95% confidence intervals, SHR; sub-distribution hazards ratios from competing risk models, HR; Hazard Ratio from Cox Proportion hazard regression models.

Supplement Table 6. Diagnosis assigned at death (all inpatient deaths).

**Diagnosis**	**Malaria Parasitaemia**					**Anaemia**					**All (n=381)**
	None (n=313)	Low (n=15)	Medium (n=18)	High (n=14)	Not available (n=21)	None (n=47)	Mild (n=59)	Moderate (n=170)	Severe (n=88)	Not available (n=17)	
LRTI	74 (24)	0	0	0	4 (19)	7 (15)	12 (20)	44 (26)	14 (16)	1 (5.9)	78 (20)
Malnutrition	56 (18)	1 (6.7)	0	1 (7.1)	1 (4.8)	5 (11)	8 (14)	29 (17)	16 (18)	1 (5.9)	59 (15)
Malaria	6 (1.9)	9 (60)	16 (89)	11 (79)	1 (4.8)	3 (6.4)	2 (3.4)	19 (11)	18 (20)	1 (5.9)	43 (11)
Heart disease	28 (8.9)	1 (6.7)	0	0	1 (4.8)	8 (17)	6 (10)	13 (7.6)	2 (2.3)	1 (5.9)	30 (7.9)
Septicaemia	25 (8.0)	2 (13)	0	0	3 (14)	0	8 (14)	15 (8.8)	4 (4.5)	3 (18)	30 (7.9)
Gastroenteritis	25 (8.0)	0	0	0	4 (19)	5 (11)	8 (14)	11 (6.5)	1 (1.1)	4 (24)	29 (7.6)
Anaemia	14 (4.5)	0	2 (11)	2 (14)	2 (9.5)	0	1 (1.7)	0	18 (20)	1 (5.9)	20 (5.2)
Encephalopathy	15 (4.8)	0	0	0	2 (9.5)	4 (8.5)	3 (5.1)	7 (4.1)	1 (1.1)	2 (12)	17 (4.5)
Immune disorder	9 (2.9)	0	0	0	2 (9.5)	0	0	8 (4.7)	2 (2.3)	1 (5.9)	11 (2.9)
Meningitis	9 (2.9)	0	0	0	0	2 (4.3)	1 (1.7)	6 (3.5)	0	0	9 (2.4)
Burns	7 (2.2)	0	0	0	0	4 (8.5)	3 (5.1)	0	0	0	7 (1.8)
Sickle cell disease	6 (1.9)	0	0	0	0	0	0	1 (0.6)	5 (5.7)	0	6 (1.6)
Congenital abnormalities	7 (2.2)	0	0	0	0	2 (4.3)	1 (1.7)	2 (1.2)	2 (2.3)	0	7 (1.8)
Hydrocephalus	5 (1.6)	0	0	0	0	1 (2.1)	1 (1.7)	2 (1.2)	1 (1.1)	0	5 (1.3)
Febrile convulsions	3 (1.0)	0	0	0	0	0	0	3 (1.8)	0	0	3 (0.8)
Cerebral palsy	3 (1.0)	0	0	0	0	0	0	1 (0.6)	2 (2.3)	0	3 (0.8)
Acute abdominal/obstruction	3 (1.0)	0	0	0	0	1 (2.1)	0	2 (1.2)	0	0	3 (0.8)
Pulmonary tuberculosis	3 (1.0)	0	0	0	0	2 (4.3)	0	0	1 (1.1)	0	3 (0.8)
Trauma/fractures	1 (0.3)	2 (13)	0	0	0	1 (2.1)	1 (1.7)	1 (0.6)	0	0	3 (0.8)
Poisoning	3 (1.0)	0	0	0	0	0	1 (1.7)	2 (1.2)	0	0	3 (0.8)
Epilepsy	2 (0.6)	0	0	0	0	1 (2.1)	0	1 (0.6)	0	0	2 (0.5)
Unclassified diseases	6 (1.9)	0	0	0	1 (4.8)	0	2 (3.4)	2 (1.2)	1 (1.1)	2 (12)	7 (1.8)
Others*	3 (1.0)	0	0	0	0	1 (2.1)	1 (1.7)	1 (0.6)	0	0	3 (0.8)

LRTI; Lower respiratory tract infection, *Asthma-1, Renal failure-1, and Jaundice -1, Low malaria parasitaemia; <2500/ul, medium malaria parasitaemia; 2500 to 250000/ul, High malaria parasitaemia; >250000/ul, no anaemia; haemoglobin ≥11g/dl, mild anaemia; haemoglobin 10 to 10.9 g/dl, moderate anaemia; haemoglobin 7 to 9.9 g/dl, severe anaemia; haemoglobin <7g/dl.

Supplement Table 7. Leading diagnoses assigned at discharge and their case fatalities.

Diagnosis (N=9431)	No (%)	Inpatient deaths N (%)
Severe pneumonia	2924 (31)	209 (7.2)
Gastroenteritis	1856 (20)	111 (6.0)
Malaria (presence of parasitaemia)	1369 (15)	47 (3.4)
Severe anaemia	1140 (12)	88 (7.7)
Severe acute malnutrition (SAM)	1071 (11)	154 (14)
Sickle cell	414 (4.4)	8 (1.9)
Epilepsy	267 (2.8)	4 (1.5)
HIV infected (positive rapid test)	267 (2.8)	38 (14)

Some children had more than one diagnosis, Diagnoses were assigned at discharge or death.

Supplement Table 8. Timing of post-discharge deaths among children admitted with anaemia and Malaria parasitaemia.

Anaemia status	Timing of post-discharge deaths			Within one-year
	Within 30 days	Within 90 days	Within 180 days	
None	11 (15.3)	20 (14.8)	29 (16.1)	36 (15.8)
Mild	9 (12.5)	14 (10.4)	22 (12.2)	31 (13.6)
Moderate	34 (47.2)	69 (51.1)	86 (47.8)	104 (45.6)
Severe	15 (20.8)	24 (17.8)	33 (18.3)	45 (19.7)
Not available	3 (4.2)	8 (5.9)	10 (5.6)	12 (5.3)
Malaria parasitaemia				
None	67 (93.1)	124 (91.9)	165 (91.7)	207 (90.8)
Low	0	0	1 (0.6)	2 (0.9)
Medium	1 (1.4)	2 (1.5)	3 (1.7)	6 (2.6)
High	1 (1.4)	2 (1.5)	2 (1.1)	2 (0.9)
Not available	3 (4.2)	7 (5.2)	9 (5.0)	11 (4.8)
Overall (everyone)	72 (32)	135 (57)	180 (79)	228

Supplement Table 9. Diagnosis assigned at death (post-discharge deaths at KCH).

**Diagnosis**	**Malaria Parasitaemia**					**Anaemia**					**All (n=97)**
	None (n=86)	Low (n=1)	Medium (n=2)	High (n=0)	Not tested (n=8)	None (n=18)	Mild (n=15)	Moderate (n=41)	Severe (n=15)	Not tested (n=8)	
LRTI	29 (34)	0	2 (100)	0	1 (13)	6 (33)	7 (47)	15 (37)	3 (20)	1 (13)	32 (33)
URTI	1 (1.2)	0	0	0	1 (13)	0	0	1 (2.4)	0	1 (13)	2 (2.1)
Malnutrition	17 (20)	0	0	0	0	3 (17)	3 (20)	9 (22)	2 (13)	0	17 (18)
Malaria	0	1 (100)	0	0	0	0	0	0	1 (6.7)	0	1 (1.0)
Heart disease	7 (8.1)	0	0	0	1 (13)	3 (17)	1 (6.7)	3 (7.3)	0	1 (13)	8 (8.2)
Septicaemia/sepsis	1 (1.2)	0	0	0	1 (13)	0	0	1 (2.4)	0	1 (13)	2 (2.1)
Gastroenteritis	3 (3.5)	0	0	0	1 (13)	2 (11)	0	1 (2.4)	0	1 (13)	4 (4.1)
Anaemia	6 (7.0)	0	0	0	0	0	0	2 (4.9)	4 (27)	0	6 (6.2)
Encephalopathy	1 (1.2)	0	0	0	0	0	0	1 (2.4)	0	0	1 (1.0)
Epilepsy	5 (5.8)	0	0	0	0	1 (5.6)	1 (6.7)	3 (7.3)	0	0	5 (5.2)
Immunosuppression	2 (2.3)	0	0	0	0	0	1 (6.7)	0	1 (6.7)	0	2 (2.1)
Meningitis	1 (1.2)	0	0	0	0	0	0	1 (2.4)	0	0	1 (1.0)
Burns	1 (1.2)	0	0	0	0	0	1 (6.7)	0	0	0	1 (1.0)
Sickle cell disease	4 (4.7)	0	0	0	0	0	0	0	4 (27)	0	4 (4.1)
Congenital abnormalities	1 (1.2)	0	0	0	2 (25)	1 (5.6)	0	0	0	2 (25)	3 (3.1)
Hydrocephalus	2 (2.3)	0	0	0	0	0	1 (6.7)	1 (2.4)	0	0	2 (2.1)
Febrile convulsions	1 (1.2)	0	0	0	0	1 (5.6)	0	0	0	0	1 (1.0)
Cerebral palsy	1 (1.2)	0	0	0	0	0	0	1 (2.4)	0	0	1 (1.0)
Acute flaccid paralysis	1 (1.2)	0	0	0	0	1 (5.6)	0	0	0	0	1 (1.0)
Cellulitis/Pyomyositis	1 (1.2)	0	0	0	1 (13)	0	0	1 (2.4)	0	1 (13)	2 (2.1)
Viral hepatitis	1 (1.2)	0	0	0	0	0	0	1 (2.4)	0	0	1 (1.0)

LRTI; Lower respiratory tract infection, URTI; Upper respiratory tract infection, Low malaria parasitaemia; <2500/ul, medium malaria parasitaemia; 2500 to 250000/ul, High malaria parasitaemia; >250000/ul, no anaemia; haemoglobin ≥11g/dl, mild anaemia; haemoglobin 10 to 10.9 g/dl, moderate anaemia; haemoglobin 7 to 9.9 g/dl, severe anaemia; haemoglobin <7g/dl.

Supplement Table 10. Diagnosis assigned at death (post-discharge deaths outside KCH assigned through verbal autopsy in the community).

**Diagnosis**	**Malaria Parasitaemia**					**Anaemia**					**All (n=131)**
	None (n=121)	Low (n=1)	Medium (n=4)	High (n=2)	Not tested (n=3)	None (n=18)	Mild (n=16)	Moderate (n=63)	Severe (n=30)	Not tested (n=4)	
Acid exposure to smoke fire & flame	1 (0.8)	0	0	0		1 (5.6)	0	0	0	0	1 (0.76)
Acute abdomen	5 (4.1)	0	0	1 (50)	0	2 (11)	1 (6.3)	1 (1.6)	2 (6.7)	0	6 (4.6)
Acute respiration infection	36 (30)	0	1 (25)	1 (50)	0	6 (33)	7 (44)	17 (27)	8 (27)	0	38 (29)
Congenital malformation	1 (0.83)	0	0	0	0	0	0	1 (1.6)	0	0	1 (0.76)
Diabetes mellitus	1 (0.83)	0	0	0	0	1 (5.6)	0	0	0	0	1 (0.76)
Diarrhoeal disease	4 (3.3)	0	0	0	0	1 (5.6)	0	3 (4.8)	0	0	4 (3.1)
Epilepsy	1 (0.83)	0	0	0	0	1 (5.6)	0	0	0	0	1 (0.76)
HIV/AIDS related	33 (27)	0	0	0	0	2 (11)	1 (6.3)	20 (32)	8 (27)	2 (50)	33 (25)
Malaria	6 (4.9)	1 (100)	1 (25)	0	0	0	3 (19)	2 (3.2)	3 (10)	0	8 (6.1)
Measles	1 (0.83)	0	0	0	0	0	0	1 (1.6)	0	0	1 (0.76)
Meningitis & encephalitis	1 (0.83)	0	0	0	0	0	0	1 (1.6)	0	0	1 (0.76)
Unspecified cardiac disease	0	0	1 (25)	0	0	0	0	1 (1.6)	0	0	1 (0.76)
Unspecified infectious disease	3 (2.5)	0	0	0	0	1 (5.6)	0	1 (1.6)	1 (3.3)	0	3 (2.3)
Road accident	1 (0.83)	0	0	0	1 (33)	1 (5.6)	0	0	0	1 (25)	2 (1.5)
Sepsis	1 (0.83)	0	0	0	0	0	0	0	1 (3.3)	0	1 (0.76)
Sickle cell disease with crisis	2 (1.7)	0	0	0	1 (33)	0	0	1 (1.6)	1 (3.3)	1 (25)	3 (2.3)
Verbal autopsy not conducted	24 (20)	0	1 (25)	0	1 (33)	2 (11)	4 (25)	14 (22)	6 (20)	0	26 (20)

Low malaria parasitaemia; <2500/ul, medium malaria parasitaemia; 2500 to 250000/ul, High malaria parasitaemia; >250000/ul, no anaemia; haemoglobin ≥11g/dl, mild anaemia; haemoglobin 10 to 10.9 g/dl, moderate anaemia; haemoglobin 7 to 9.9 g/dl, severe anaemia; haemoglobin <7g/dl.

Supplement Table 11. The effects of anaemia levels on post-discharge mortality among children with and without a comorbidity of and level of malaria parasitaemia.

	**Post-discharge mortality**				
	Deaths (N=228)	Univariate model		Multivariable model	
		Crude HR (95% CI)	P-value	Adjusted HR (95% CI)*	P-value
**Without Malaria**					
Anaemia status					
None	36 (2.5)	Reference		Reference	
Mild	33 (2.0)	0.80 (0.47–1.36)	0.42	0.75 (0.45–1.24)	0.26
Moderate	103 (3.5)	1.60 (1.03–2.49)	0.04	1.15 (0.76–1.74)	0.51
Severe	39 (6.4)	3.57 (1.93–6.63)	<0.0001	2.39 (1.34–4.26)	0.003
Not available	2 (2.1)	0.93 (0.19–4.50)	0.93	0.67 (0.15–3.00)	0.61
**With Malaria**					
Anaemia status					
None	1 (0.6)	Reference		Reference	
Mild	0	-		-	
Moderate	4 (0.8)	1.45 (0.16–12.9)	0.95	0.34 (0.03–4.35)	0.41
Severe	6 (1.8)	3.22 (0.39–26.8)	0.28	1.87 (0.18–19.3)	0.60
Not available	0	-		-	

*adjusted for age, HIV, malnutrition, sickle cell, cerebral palsy, heart disease, known epilepsy, bacteraemia, season, year of admission, KHDSS area and blood transfusion, HR; Hazard Ratio from multilevel mixed-effects parametric survival regression analysis with Weibull distribution.

Supplement Table 12. The effect of admission with various levels of anaemia on inpatient deaths and all-cause post-discharge mortality (full multivariable models).

	**Inpatient mortality**		**One-year post-discharge mortality**	
	aSHR (95% CI)*	P-value	aHR (95% CI)*	P-value
Anaemia status				
None	Reference		Reference	
Mild	1.05 (0.92–1.19)	0.47	0.60 (0.35–1.03)	0.07
Moderate	1.38 (1.12–1.69)	0.002	1.06 (0.69–1.62)	0.80
Severe	1.94 (1.71–2.20)	<0.001	1.94 (1.11–3.40)	0.02
Not available	1.83 (1.52–2.21)	<0.001	1.46 (0.63–3.40)	0.38
Age in months (log transformed)	0.95 (0.92–0.99)	0.005	0.71 (0.59–0.85)	<0.001
Nutritional status				
Not wasted	Reference		Reference	
Moderate wasted	1.98 (1.84–2.13)	<0.001	2.48 (1.60–3.84)	<0.001
Severe wasted	3.65 (3.44–3.98)	<0.001	7.55 (4.67–12.2)	<0.001
Missing MUAC	7.29 (6.99–7.59)	<0.001	8.11 (2.19–30.1)	0.002
KHDSS region				
North of KCH	Reference		Reference	
Kilifi Township	0.78 (0.74–0.82)	<0.001	0.77 (0.50–1.19)	0.24
South of KCH	0.98 (0.88–1.09)	0.74	1.04 (0.74–1.47)	0.80
Season of the year				
Dry	Reference		Reference	
Rainy	0.97 (0.86–1.09)	0.62	1.24 (0.92–1.66)	0.16
Year of admission				
2010	Reference		Reference	
2011	1.17 (1.03–1.33)	0.01	1.03 (0.57–1.85)	0.93
2012	1.36 (1.17–1.58)	<0.001	0.91 (0.47–1.74)	0.77
2013	1.93 (1.38–2.69)	<0.001	1.99 (1.07–3.69)	0.03
2014	1.83 (1.46–2.29)	<0.001	1.61 (0.88–3.00)	0.12
2015	1.59 (1.32–1.91)	<0.001	1.75 (0.97–3.15)	0.06
2016	1.63 (1.45–1.84)	0.001	1.64 (0.85–3.18)	0.14
2017	3.53 (2.57–4.87)	<0.001	2.20 (1.07–4.52)	0.03
2018	1.80 (1.52–2.11)	<0.001	2.01 (1.11–3.66)	0.02
2019	2.14 (1.85–2.46)	<0.001	1.38 (0.72–2.66)	0.34
Clinical presentation/diagnosis				
HIV status				
Negative	Reference		Reference	
Infected	2.03 (1.93–2.14)	<0.001	4.96 (2.65–9.27)	<0.001
Unknown	1.41 (1.37–1.46)	<0.001	1.45 (0.92–2.27)	0.11
Sickle cell disease	0.40 (0.24–0.67)	<0.001	1.33 (0.54–3.26)	0.53
Cerebral palsy	1.65 (1.31–2.08)	<0.001	2.65 (0.96–7.37)	0.06
Heart disease	3.74 (2.92–4.77)	<0.001	10.9 (5.43–21.9)	<0.001
Epilepsy	0.46 (0.31–0.68)	<0.001	3.37 (1.48–7.66)	0.004
Bacteraemia	2.44 (2.01–2.95)	<0.001	1.21 (0.68–2.16)	0.52
Transfused before discharge	2.00 (1.72–2.33)	<0.001	1.21 (0.65–2.25)	0.55

*SHR; sub-distribution hazards ratios from competing risk models, HR; Hazard Ratio from multilevel mixed-effects parametric survival regression analysis with Weibull distribution.

Supplement Table 13. The effect of admission with Malaria Parasitaemia on inpatient deaths and all-cause post-discharge mortality (full multivariable models).

	**Inpatient mortality**		**One-year post-discharge mortality**	
	aSHR (95% CI)*	P-value	aHR (95% CI)*	P-value
Malaria parasitaemia				
None	Reference		Reference	
Low	1.20 (1.13–1.27)	<0.001	0.26 (0.06–1.10)	0.07
Medium	0.67 (0.63–0.72)	<0.001	0.35 (0.15–0.84)	0.02
High	2.37 (2.00–2.80)	<0.001	0.53 (0.12–2.23)	0.38
Not available	2.21 (1.69–2.90)	<0.001	1.33 (0.60–2.94)	0.48
Age in months (log transformed)	0.97 (0.94–1.01)	0.15	0.79 (0.66–0.94)	0.009
**Nutritional status**				
Not wasted	Reference		Reference	
Moderate wasted	2.11 (1.94–2.29)	<0.001	2.48 (1.61–3.83)	<0.001
Severe wasted	3.99 (3.62–4.39)	<0.001	7.24 (4.43–11.8)	<0.001
Missing MUAC	6.37 (6.17–6.59)	<0.001	8.07 (2.20–29.6)	0.002
KHDSS region				
North of KCH	Reference		Reference	
Kilifi Township	0.77 (0.73–0.81)	<0.001	0.79 (0.52–1.20)	0.27
South of KCH	0.99 (0.91–1.09)	0.88	1.22 (0.87–1.71)	0.24
Season of the year				
Dry	Reference		Reference	
Rainy	0.96 (0.85–1.08)	0.50	1.18 (0.88–1.58)	0.26
Year of admission				
2010	Reference		Reference	
2011	1.18 (1.02–1.37)	0.03	1.02 (0.57–1.80)	0.95
2012	1.37 (1.20–1.57)	<0.001	0.88 (0.46–1.67)	0.69
2013	1.99 (1.46–2.72)	<0.001	1.96 (1.07–3.59)	0.03
2014	1.92 (1.55–2.37)	<0.001	1.61 (0.89–2.94)	0.12
2015	1.66 (1.39–1.98)	<0.001	1.78 (1.00–3.17)	0.05
2016	1.66 (1.44–1.92)	<0.001	1.58 (0.83–3.03)	0.16
2017	3.58 (2.50–5.14)	<0.001	2.10 (1.04–4.24)	0.04
2018	1.79 (1.55–2.07)	<0.001	1.80 (1.00–3.23)	0.05
2019	2.11 (1.89–2.35)	<0.001	1.32 (0.69–2.51)	0.39
Clinical presentation/diagnosis				
HIV status				
Negative	Reference		Reference	
Infected	2.03 (1.93–2.14)	<0.001	4.50 (2.41–8.38)	<0.001
Unknown	1.39 (1.34–1.43)	<0.001	1.49 (0.95–2.32)	0.08
Sickle cell disease	0.48 (0.28–0.82)	0.007	1.32 (0.54–3.23)	0.54
Cerebral palsy	1.60 (1.26–2.03)	<0.001	2.36 (0.88–6.32)	0.09
Heart disease	3.33 (2.56–4.32)	<0.001	9.63 (4.79–19.3)	<0.001
Epilepsy	0.42 (0.27–0.63)	<0.001	2.68 (1.20–5.99)	0.02
Bacteraemia	2.54 (2.14–3.01)	<0.001	1.24 (0.70–2.19)	0.45
Transfused before discharge	2.52 (1.15–3.02)	<0.001	2.16 (1.21–3.86)	0.009

*SHR; sub-distribution hazards ratios from competing risk models, HR; Hazard Ratio from multilevel mixed-effects parametric survival regression analysis with Weibull distribution.

Supplementary Figures

Supplement Figure 4. Stacked bar chart of a) Malaria parasitaemia and b) Anaemia by age groups (age in months).

A

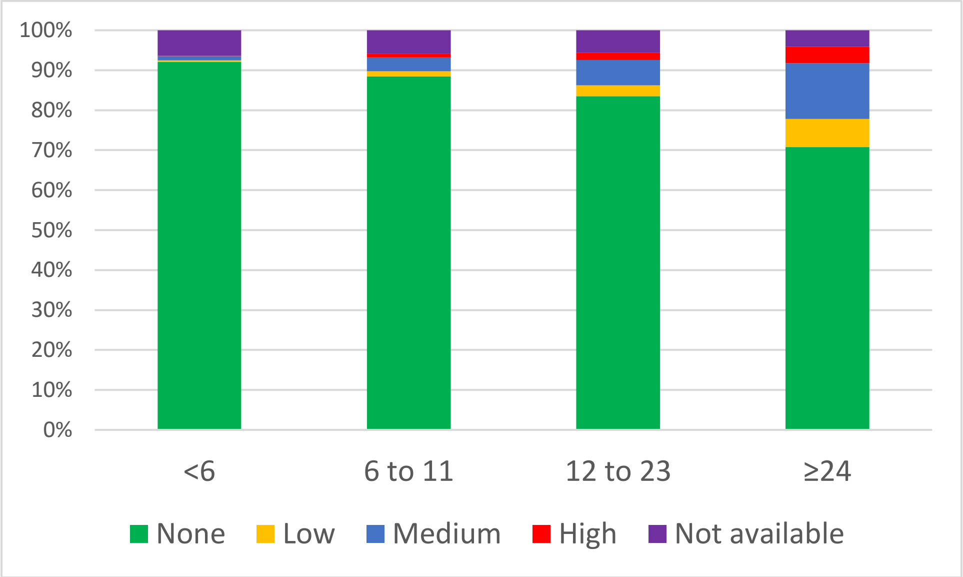

B

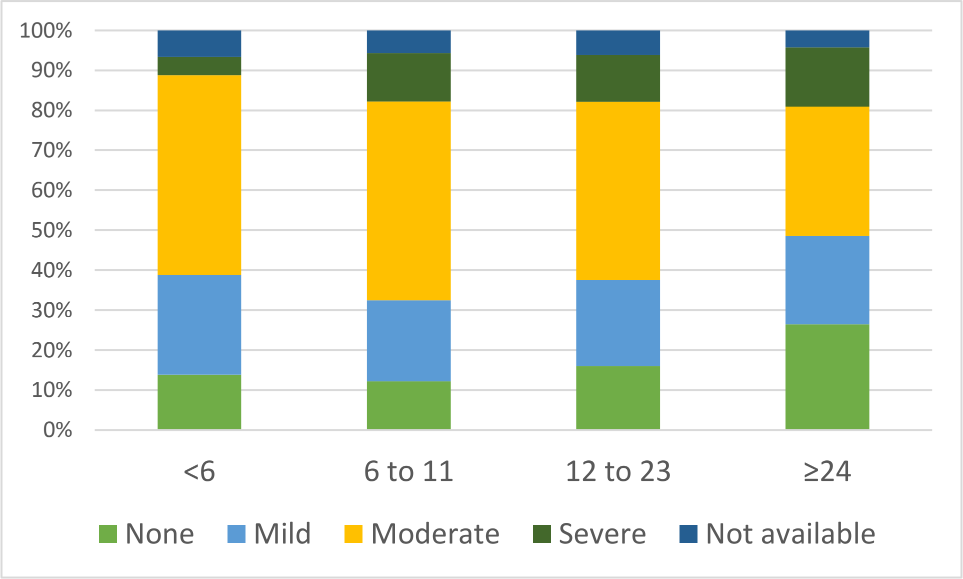

Supplement Figure 5. Effect of blood transfusion on risk of post-discharge deaths among admissions with anaemia.

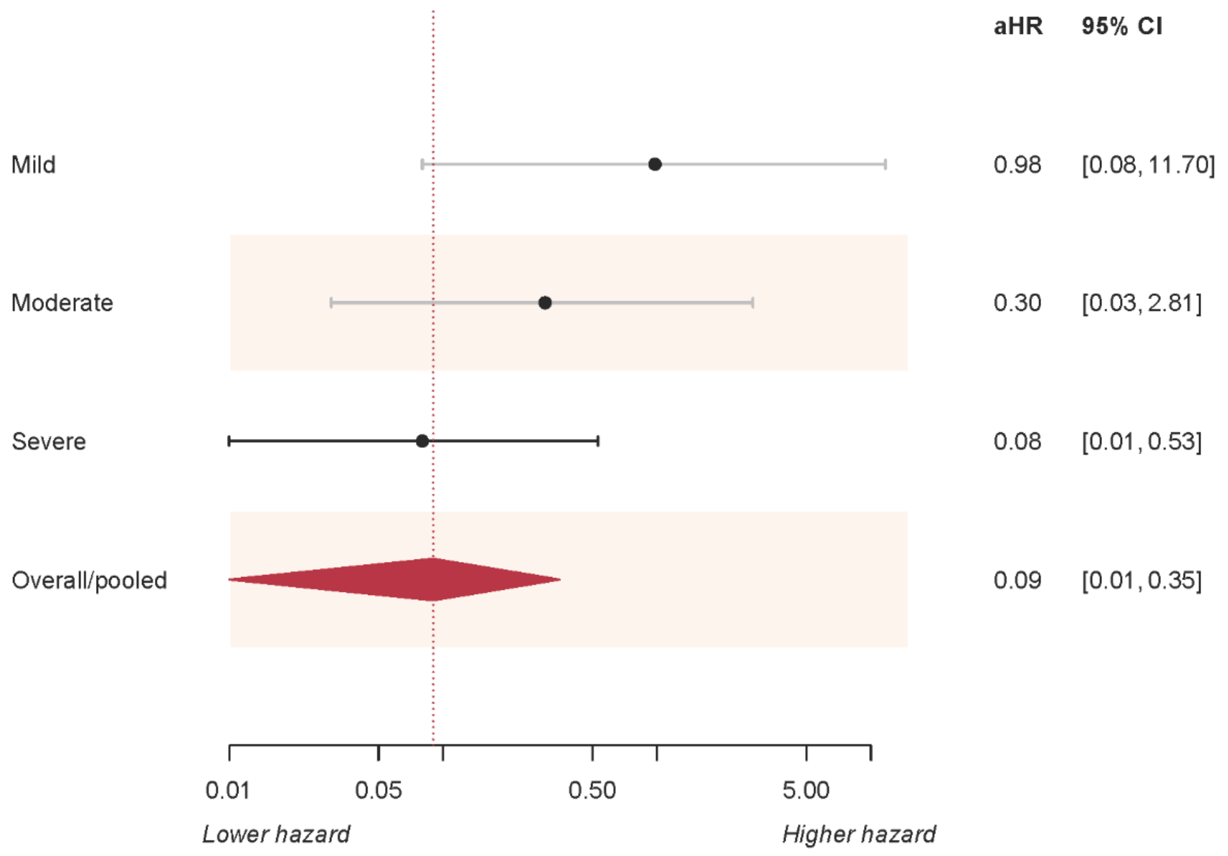

STROBE Statement

STROBE Statement—Checklist of items that should be included in reports of ***cohort studies***

	Item No	Recommendation
**Title and abstract**	1	(*a*) Indicate the study's design with a commonly used term in the title or the abstract (**P1**)
		(*b*) Provide in the abstract an informative and balanced summary of what was done and what was found (**Abstract P2**)
**Introduction**		
Background/rationale	2	Explain the scientific background and rationale for the investigation being reported (**Background P3/4**)
Objectives	3	State specific objectives, including any prespecified hypotheses **(Background P4**)
**Methods**		
Study design	4	Present key elements of study design early in the paper (**Methods P5**)
Setting	5	Describe the setting, locations, and relevant dates, including periods of recruitment, exposure, follow-up, and data collection (**Methods P4/5**)
Participants	6	(*a*) Give the eligibility criteria, and the sources and methods of selection of participants. Describe methods of follow-up (**Methods P5 (Study Population**))
		(*b*) For matched studies, give matching criteria and number of exposed and unexposed (**N/a**)
Variables	7	Clearly define all outcomes, exposures, predictors, potential confounders, and effect modifiers. Give diagnostic criteria, if applicable (**Methods P6/7**)
Data sources/ measurement	8*	For each variable of interest, give sources of data and details of methods of assessment (measurement). Describe comparability of assessment methods if there is more than one group (**Methods P5/6**)
Bias	9	Describe any efforts to address potential sources of bias (**Methods-statistical methods P7/8**)
Study size	10	Explain how the study size was arrived at (**Methods P6**)
Quantitative variables	11	Explain how quantitative variables were handled in the analyses. If applicable, describe which groupings were chosen and why (**Methods P6/7**)
Statistical methods	12	(*a*) Describe all statistical methods, including those used to control for confounding (**Methods-statistical methods P7/8**)
		(*b*) Describe any methods used to examine subgroups and interactions (**Methods P8**)
		(*c*) Explain how missing data were addressed (**Methods P7**)

		(*d*) If applicable, explain how loss to follow-up was addressed **Methods P7)**
		(*e*) Describe any sensitivity analyses (**Methods P8**)
**Results**		
Participants	13*	(a) Report numbers of individuals at each stage of study—eg numbers potentially eligible, examined for eligibility, confirmed eligible, included in the study, completing follow-up, and analysed (**Results P9 and Figure 1)**
		(b) Give reasons for non-participation at each stage (**Results P9 and Figure 1)**
		(c) Consider use of a flow diagram (**Flow chart Figure 1**)
Descriptive data	14*	(a) Give characteristics of study participants (eg demographic, clinical, social) and information on exposures and potential confounders (**Results P9 and Table 1**)
		(b) Indicate number of participants with missing data for each variable of interest (**Figure 1**)
		(c) Summarise follow-up time (eg, average and total amount) (**Results P11 and Appendix P 9**)
Outcome data	15*	Report numbers of outcome events or summary measures over time (**Results P 10/11, Table 2**)
Main results	16	(*a*) Give unadjusted estimates and, if applicable, confounder-adjusted estimates and their precision (eg, 95% confidence interval). Make clear which confounders were adjusted for and why they were included (**Results P 10/11, Table 2**)
		(*b*) Report category boundaries when continuous variables were categorized (**Table 1**)
		(*c*) If relevant, consider translating estimates of relative risk into absolute risk for a meaningful time period (**N/A**)
Other analyses	17	Report other analyses done—eg analyses of subgroups and interactions, and sensitivity analyses (**Results P12**)
**Discussion**		
Key results	18	Summarise key results with reference to study objectives (**Discussion P13**)
Limitations	19	Discuss limitations of the study, taking into account sources of potential bias or imprecision. Discuss both direction and magnitude of any potential bias (**Discussion P15**)
Interpretation	20	Give a cautious overall interpretation of results considering objectives, limitations, multiplicity of analyses, results from similar studies, and other relevant evidence (**Discussion P13/15**)
Generalisability	21	Discuss the generalisability (external validity) of the study results (**Discussion P 14/16**)

**Other information**		
Funding	22	Give the source of funding and the role of the funders for the present study and, if applicable, for the original study on which the present article is based (**Funding P17**)

*Give information separately for exposed and unexposed groups.

Note: An Explanation and Elaboration article discusses each checklist item and gives methodological background and published examples of transparent reporting. The STROBE checklist is best used in conjunction with this article (freely available on the Web sites of PLoS Medicine at http://www.plosmedicine.org/, Annals of Internal Medicine at http://www.annals.org/, and Epidemiology at http://www.epidem.com/). Information on the STROBE Initiative is available at http://www.strobe-statement.org.

Author Reflexivity Statement

1. **How does this study address local research and policy priorities?**

Anaemia and malaria are leading causes of paediatric hospitalisation and inpatient mortality in sub-Saharan Africa including in Kenya. Mortality following discharge from hospital in resource-poor countries has been recognized as a significant contributor to childhood mortality. However, there is limited empirical data on survival following hospital admission distinguishing the effects of anaemia and malaria parasitaemia and this paper seeks to fill this gap.

2. **How were local researchers involved in study design?**

The study was conceived, designed and executed by local researchers (MMN, MKM, AK). The data for the study were collected and curated by local researchers (NM, NO, AN, CO). The statistical analysis and writing of the first draft of manuscript was conducted by MMN.

3. **How has funding been used to support the local research team?**

The Kilifi Health Demographic Surveillance System (KHDSS) and the Kilifi County Hospital ward surveillance are funded by the Wellcome Trust which pays salaries to the local research team. Various grants were used to pay salaries for all local co-authors.

4. **How are research staff who conducted data collection acknowledged?**

The study data were collected by NM and CO and curated by NO and AN who are co-authors in this manuscript.

5. **Do all members of the research partnership have access to study data?**

The study raw data were accessible by MMN, NO, AN, RWS and JAB.

6. **How was data used to develop analytical skills within the partnership?**

Data analysis was conducted by MMN with supervision and guidance from RWS and JAB. Meetings were conducted with the other co-authors to interpret findings and make analytic adjustments as required.

7. **How have research partners collaborated in interpreting study data?**

MMN, AK, RWS and JAB were involved in interpretation of preliminary findings through discussions and meetings. All co-authors reviewed and commented on study results.

8. **How were research partners supported to develop writing skills?**
MMN was supported by the senior co-authors (RWS and JAB) to develop and refine writing skills.

9. **How will research products be shared to address local needs?**

The study findings will be presented to the Kenya Paediatric Association conference and KEMRI Annual Scientific conference. The paper will be published in a peer-reviewed journal making the findings available to both local and international researchers and policymakers.

10. **How is the leadership, contribution and ownership of this work by LMIC researchers recognized within the authorship?**

MMN who was key in designing the study and writing the manuscript, is the first author. Out of the 12 authors, the first seven are local researchers.

11. **How have early career researchers across the partnership been included within the authorship team?**

MMN who is the first author, CO and AK are early career researchers.

12. **How has gender balance been addressed within the authorship?**

Five (MKM, NM, CO, AK and KM) are female researchers while seven were males.

13. **How has the project contributed to training of LMIC researchers?**

MMN, MKM, CO, AK were all supervised for their PhD and postdoctoral projects by JAB and RWS.

14. **How has the project contributed to improvements in local infrastructure?**

This study was a secondary analysis of collected data, it has not directly contributed to improvements in local infrastructure.

15. **What safeguarding procedures were used to protect local study participants and researchers?**

The study was approved by local ethical body to use already collected data. We used secondary anonymized data. Out of the 12 co-authors in the manuscript, seven were local researchers including those collecting data in the ward.

References

1. Ngari MM, Obiero C, Mwangome MK, et al. Mortality during and following hospital admission among school-aged children: a cohort study. *Wellcome Open Res* 2020; **5**: 234.
2. Talbert A, Ngari M, Bauni E, et al. Mortality after inpatient treatment for diarrhea in children: a cohort study. *BMC Med* 2019; **17**(1): 20.
3. Talbert A, Ngari M, Obiero C, et al. Trends in inpatient and post-discharge mortality among young infants admitted to Kilifi County Hospital, Kenya: a retrospective cohort study. *BMJ Open* 2023; **13**(1): e067482.
4. Mogeni P, Williams TN, Fegan G, et al. Age, Spatial, and Temporal Variations in Hospital Admissions with Malaria in Kilifi County, Kenya: A 25-Year Longitudinal Observational Study. *PLoS Med* 2016; **13**(6): e1002047.
5. Kamau A, Mtanje G, Mataza C, et al. Malaria infection, disease and mortality among children and adults on the coast of Kenya. *Malar J* 2020; **19**(1): 210.
6. World Health Organization (WHO). Pocket Book of Hospital Care for Children: Guidelines for the Management of Common Childhood Illnesses. 2nd ed. Geneva; 2013.
7. Mwangome MK, Fegan G, Prentice AM, Berkley JA. Are diagnostic criteria for acute malnutrition affected by hydration status in hospitalized children? A repeated measures study. *Nutr J* 2011; **10**: 92.
8. Ndila C, Bauni E, Mochamah G, et al. Causes of death among persons of all ages within the Kilifi Health and Demographic Surveillance System, Kenya, determined from verbal autopsies interpreted using the InterVA-4 model. *Glob Health Action* 2014; **7**: 25593.
